# Genomic tools development for *Aquilegia*: construction of a BAC-based physical map

**DOI:** 10.1186/1471-2164-11-621

**Published:** 2010-11-08

**Authors:** Guang-Chen Fang, Barbara P Blackmon, David C Henry, Margaret E Staton, Christopher A Saski, Scott A Hodges, Jeff P Tomkins, Hong Luo

**Affiliations:** 1 Department of Genetics and Biochemistry, Clemson University, 100 Jordan Hall, Clemson, SC 29634, USA; 2 Clemson University Genomics Institute, Clemson University, Biosystems Research Complex, 51 New Cherry Street, Clemson, SC 29634, USA; 3 Department of Ecology, Evolution, and Marine Biology, University of California, Santa Barbara, CA 93106, USA

## Abstract

**Background:**

The genus *Aquilegia*, consisting of approximately 70 taxa, is a member of the basal eudicot lineage, Ranuculales, which is evolutionarily intermediate between monocots and core eudicots, and represents a relatively unstudied clade in the angiosperm phylogenetic tree that bridges the gap between these two major plant groups. *Aquilegia *species are closely related and their distribution covers highly diverse habitats. These provide rich resources to better understand the genetic basis of adaptation to different pollinators and habitats that in turn leads to rapid speciation. To gain insights into the genome structure and facilitate gene identification, comparative genomics and whole-genome shotgun sequencing assembly, BAC-based genomics resources are of crucial importance.

**Results:**

BAC-based genomic resources, including two BAC libraries, a physical map with anchored markers and BAC end sequences, were established from *A. formosa*. The physical map was composed of a total of 50,155 BAC clones in 832 contigs and 3939 singletons, covering 21X genome equivalents. These contigs spanned a physical length of 689.8 Mb (~2.3X of the genome) suggesting the complex heterozygosity of the genome. A set of 197 markers was developed from ESTs induced by drought-stress, or involved in anthocyanin biosynthesis or floral development, and was integrated into the physical map. Among these were 87 genetically mapped markers that anchored 54 contigs, spanning 76.4 Mb (25.5%) across the genome. Analysis of a selection of 12,086 BAC end sequences (BESs) from the minimal tiling path (MTP) allowed a preview of the *Aquilegia *genome organization, including identification of transposable elements, simple sequence repeats and gene content. Common repetitive elements previously reported in both monocots and core eudicots were identified in *Aquilegia *suggesting the value of this genome in connecting the two major plant clades. Comparison with sequenced plant genomes indicated a higher similarity to grapevine (*Vitis vinifera*) than to rice and *Arabidopsis *in the transcriptomes.

**Conclusions:**

The *A. formosa *BAC-based genomic resources provide valuable tools to study *Aquilegia *genome. Further integration of other existing genomics resources, such as ESTs, into the physical map should enable better understanding of the molecular mechanisms underlying adaptive radiation and elaboration of floral morphology.

## Background

Recent progress in genomic research using the model species *A. thaliana *and crop species, such as rice, maize, sorghum and tomato, has dramatically enhanced our capacity to unravel the genetic basis of biological diversity and the evolution of complex traits and genetic pathways in plants. These include genes and pathways determining plant architecture and fruit size [1,2], flowering time [3-6], light response [7,8] and plant defence [9]. However, when studying fundamentals about how organisms have adapted to their natural environments, the information derived especially from crops is of limited application. In these species, many of the traits have undergone intensive artificial selection over the course of directed genetic improvement. In addition, these plant species are often highly inbred due to either artificial selection or self-pollination, which may lead to an increased likelihood of accumulating deleterious alleles, and consequently the loss of atypical patterns of much of the genetic variation displayed in natural systems [10,11]. It is, therefore, critical to identify and develop new model species from natural settings, with well-defined ecologies and abundant examples of adaptation to various environments to advance our understanding of plant evolution, development and ecology.

Several important factors need to be carefully weighed when choosing new model species to develop tools for genomic studies [12,13]. First, it is desirable that the new systems encompass a wide range of morphological and ecological diversity occurring in the flowering plants. This will not only allow understanding of the morphological variation in response to critical phenomena, but also facilitate investigation of the physiological adaptation to new environments. Second, with the information accumulated from the increasing number of model systems within grasses (e.g., rice, maize, sorghum, and *Brachypodium distachyon*) and core eudicots (e.g., *Arabidopsis*, Medicago, tomato, and *Mimulus*), the development of phylogenetically intermediate model systems would greatly facilitate genome comparisons among these taxa and bridge the deep evolutionary distance between the well-developed *Arabidopsis *and rice model systems [13,14]. Third, it is ideal that genetic resources developed for new model systems could be transferable to a wide-range of related species, serving to address many questions for the community at large [13].

*Aquilegia*, the columbine genus [15], is emerging as a new evolutionary genomic model in a relatively unstudied area of the plant phylogenetic tree, the basal eudicots. *Aquilegia *species are so closely related that they have been considered a species flock or syngameon [15,16]. This group was extensively studied by Verne Grant as an interesting example of recent adaptive radiation leading to rapid speciation [16]. Variation in pollinators has apparently driven the evolution of a wide variety of floral morphologies and, along with adaptation to different habitats, likely induced rapid reproductive isolation [16,17]. Approximately 23 different species in North America emerged in as little as 1-3 million years [18] resulting in little sequence variation in DNA regions such as chloroplast and rDNA [15]. Remarkably, ecologically and morphologically distinct species share the majority of sequence polymorphisms making them difficult to distinguish at the molecular level [17,19-21], which again suggests that these taxa are of very recent origin. As such, it is not surprising that it has long been known that species of *Aquilegia *are highly cross-compatible [16] which provides an opportunity for genetic studies across multiple fields such as ecology, physiology and morphology [11,16 ,22,23]. The feature of cross compatibility not only facilitates the genetic dissection of traits, but also suggests that genomic tools developed from a single species will be readily transferred to a wide range of additional species [24].

Phylogenetically, *Aquilegia *belongs to the plant family Ranunculaceae, which is a basal-eudicot lineage [23,25]. This position of being approximately equidistant, evolutionarily, from the current monocot (rice) and core-eudicot (*Arabidopsis*) model plant systems provides a unique opportunity for comparative studies among angiosperms of sequence information, genome structure, and the conservation/diversification of developmental pathways. For instance, it has been hypothesized that a whole-genome duplication occurred near the base of the eudicot lineage yet *Aquilegia*, and thus the Ranunculales, appears to have predated this event [26-28]. Moreover, *Aquilegia *possesses unusual floral morphology such as petaloid sepals, nectar spurs and a recently evolved novel floral organ, the staminodium, not available for study in current model systems [29]. These traits, along with its small genome size (~300 Mb), all support *Aquilegia *as an important new model for plant development, ecology and evolution.

Here, we report the construction of a physical map of the *A. formosa *genome using the High Information Content Fingerprinting (HICF) method [30]. A single individual from the wild was used for library construction because inbreeding depression has been found to be exceptionally strong in all species of *Aquilegia *studied to date [31]. This plant has also been used as a grandparent in a large F2 cross between *A. formosa *and *A. pubescens*, which was utilized as the tissue source for a large EST database http://compbio.dfci.harvard.edu/cgi-bin/tgi/gimain.pl?gudb=aquilegia and is being used for QTL mapping. In addition, we integrated a total of 197 markers (many derived from the EST database) by multi-dimensional pool hybridization, and produced BAC end sequences of a minimum tiling path. Thus, the physical map described here integrates across a number of studies and genomic resources for *Aquilegia*. The physical map and other information presented here will help facilitate the long-range assembly of *Aquilegia *genome sequence and fine-scale mapping of QTLs as well as comparative genomic studies. Because all species of *Aquilegia *are highly similar at the genetic level (see above), this resource should be important for genomic studies spanning the entire genus.

## Results

### BAC library characterization

Two complementary restriction derived large-insert BAC libraries of a single *A. formosa *plant were used for constructing a physical map. Tissue was collected in the wild along Bishop Creek Inyo Co., CA and sent to Amplicon Express (Pullman, WA) for library construction. This same individual *A. formosa *plant has been used as a parent to construct an *A. formosa *× *A. pubescens *F2 population for genetic mapping and EST sequencing [32]. As summarized in Table 1, the *A. formosa Hin*dIII BAC library (AF_Bb) has an estimated genome coverage of 15.2X and contains 29,568 clones with an average insert size of 144.6 kb (N = 186). The insert sizes range from 60-300 kb, and 87% of the clones have an insert size of 100 kb or larger. The library has 4.8% empty vector clones. The *A. formosa Mbo*I BAC library (AF_Bc) has an estimated genome coverage of 13.3X and contains 28,800 clones with an average insert size of 110.9 kb (N = 187). The insert size range is 35-290 kb. The library contains 59.4% clones with insert size of 100 kb or larger, and 0.5% empty vector clones. Other than these two libraries, a third BAC library was constructed from *A. coerulea *"Goldsmith" ADQ47, which is currently being used for whole genome sequencing at Joint Genome Institute (JGI) for comparative genomic studies. The library was created by partial digestion of high molecular weight genomic DNA with *Hind*III according to the procedure of Peterson *et al. *[33]. The library consisted of a total of 47,616 clones with an average insert size of 131 kb to cover 20.7 genome equivalents.

**Table 1:** Summary of BAC libraries and HICF fingerprinting of BAC clones

Library	Restriction digest	Cloning vector	Average insert size (kb)	# clones	Genome coverage	Valid bands per clone (average)	# of clones fingerprinted
AF_Bb^a^	*Hind*III	pECBAC1	144.6	29,568	15.2 X	83.7	28,728
AF_Bc^a^	*Mbo*I	pECBAC1	110.9	28,800	13.3 X	78.6	28,393
Total			128	58,368	28.5 X	81.1	57,121^b^

^a^The 2 libraries were constructed from *A. formosa*

^b^Among the 57121 clones were 50,155 clones used for FPC project

### HICF fingerprinting

A total of 28,728 and 28,393 clones from *A. formosa *AF_Bb and AF_Bc BAC libraries, respectively, was successfully fingerprinted using HICF method of Luo *et al. *[30]. The analysis resulted in an average of 83.7 and 78.6 bands per clone for the AF_Bb and AF_Bc libraries, respectively, with an overall average of 81.1 bands per clone (Table 1). Of the 57,121 successfully fingerprinted clones from both libraries, 50,155 were successfully assigned electronic fingerprints with the GenoProfiler [34] software for subsequent assembly using FPC v8.5.3 software [35]. Given 128 kb for the average insert size from both libraries, the clones included in the FPC project represent approximately 21X of *Aquilegia *genome (1N ≃ 300 Mb) (Table 2).

**Table 2:** Summary of the *Aquilegia *physical map constructed by HICF

Number of clones successfully fingerprinted	57,121
Number of clones in Physical Map	50,155
Number of clones in contigs	46,216 (92%)
Singletons	3,939
Size of contigs	
2 clones	78
3-9 clones	133
10-24 clones	141
25-49 clones	175
50-99 clones	175
100-199 clones	99
200-399 clones	27
>400	4
Number of contigs	832
Physical length of the contigs (Mb)	689.8

### Physical Map Assembly

Fingerprints from both BAC libraries were combined for contig assembly using a tolerance of 3 and a cutoff of 1e-50. The initial assembly resulted in 3,444 contigs containing 39,489 (78.7%) clones and 10,674 singletons. Eight clones were manually removed due to poor fingerprinting. The average Sulston score was 0.879. After using the DQer to break up contigs consisting of more than 10% questionable clones, a consecutive reduction in stringency at 1e-5 for each End-End and Single-End merge using the "clone plus markers (CPM)" function at a tolerance of 3 was performed to reassemble contigs. The final systematic contig assembly was calculated at a cutoff value of 1e-35. Further manual contig merges, based on the results from marker hybridization (as described in "Methods"), were conducted at a cutoff value of 1e-20 and tolerance of 3, and resulted in a total of 832 contigs consisting of 46,216 (92.1%) clones and 3,939 singletons (Table 2). A minimal tiling path (MTP) was selected using default parameters of FPC and 6,505 clones were selected for end sequencing.

### Overgo probe hybridization and marker integration

**Figure 1:**
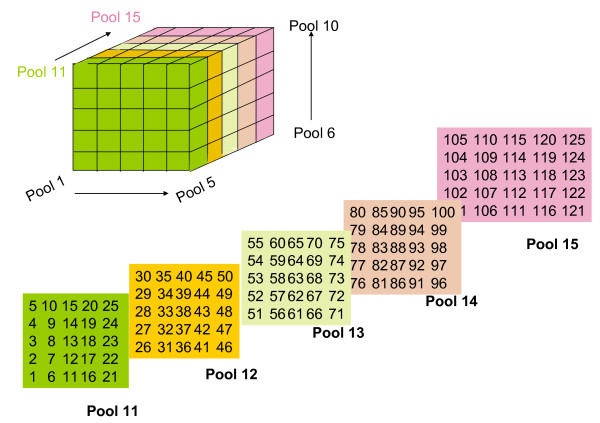
**The three-dimensional pooling strategy**. A total of 125 probes were assigned into 15 pools. Each pool contained 25 probes in a 5 × 5 format. Each probe had an unique coordinate, which can be translated from the deconvolution script.

To bridge genetic and physical maps and isolate a list of genes implicated in environmental adaptations, floral development and color for further studies, overgo probes were developed from (a) a list of markers that had been genetically mapped, (b) a list of ESTs expressed upon drought stress, (c) a list of genes potentially related to anthocyanin biosynthesis [36] and (d) a list of genes associated with flower development. Each hybridization experiment included a total of 125 markers assigned in 15 pools and each pool consisted of 25 markers (Figure 1). We considered a "hit" (i.e., positively identified BAC) as one that all three different pools containing a probe produced a clear hybridization signal. This 3-dimentional-pool hybridization approach has the advantage of reducing the number of false positive clones. Results from the first pool hybridization anchored a total of 95 drought-induced genes to the physical map with 79 markers hybridized to a single contig, 10 to two contigs, and only 6 markers to three or more contigs (Table 3). Similarly, the second pool hybridization placed 102 markers on the map and anchored a total of 65 contigs. Among these 102 markers were 87 genetically mapped markers that collectively anchored 54 contigs, covering a total of 76.4 Mb (25.5% genome) (Table 4). Furthermore, most of these markers from the second pool hybridization were mapped to separate contigs, only 14 contigs contained 2 markers, and only 1 contig had 3 markers (Table 5). All the markers, except TC32786, which failed to hybridize, were derived from genes potentially involved in anthocyanin biosynthesis and mapped to different contigs (Table 6). In summary, results from the two hybridization experiments anchored a total of 197 markers to the physical map. Among the markers were 177 markers (90%) mapped to single contigs, 12 (6%) mapped to 2 contigs, and 8 (4%) mapped to multiple (≥3) contigs. Details of all contigs are available from the WebFPC project located at http://www.genome.clemson.edu/physical_maps/aquilegia.

**Table 3:** Statistics for hybridization with drought-stress induced markers

Total number of markers used	125
Number of markers failed	30
Number of markers hybridized to single contig	79
Number of markers hybridized to two contigs	10
Number of markers hybridized to more than 2 contigs	6

**Table 4:** Summary of the contigs anchored to different linkage groups by mapped genetic markers

Linkage group	Genetic markers	**Anchored contigs**^**a**^	**Mapped contig**^**b**^	**Length (Mb)**^**c**^
LG1	9	11	7	8.7
LG2	16	16	10	13.3
LG3	14	8	8	10.7
LG4	19	12	11	15.7
LG5	7	4	4	5.1
LG6	12	9	9	14.8
LG7	10	5	5	8.1
Total	87	65	54	76.4

^a^Total number of contigs hybridized to the genetic markers on each linkage group

^b^The number of the contigs confirmed (i.e., without conflict markers) to be mapped on each linkage group

^c^Total physical length of the contigs that were confirmed to be mapped on the linkage group

**Table 5:** *Aquilegia *physical contigs mapped by two or more genetic markers from the second pool hybridization

Contig	TC Marker	Map position (Linkage group/cM)
Ctg404	TC1022, TC9132, TC15023	3/75.7 - 80.3
		
Ctg814	TC8452, TC14418	4/50.3 - 51.3
		
Ctg600	TC14475, TC14967^a^	3/64.6
Ctg1690	TC8499^a^, TC8191	2/87.7
Ctg596	TC14979, TC15808	2/33.5 - 40.3
Ctg1119	TC14131^a^, TC21922*	na
Ctg348	TC30032*, TC10108^a^	na
Ctg814	TC8452, TC14418	4/50.3 - 51.3
		
Ctg509	TC27360*, TC14816	1/12.9
		
Ctg490	TC15181^a^, TC14816	1/12.9
		
Ctg200	TC15995^a^, TC14391	2/74.7
Ctg516	TC14337^a^, TC15591	3/46.4
		
Ctg1019	TC15053, TC14816	1/98.0, 12.9
Ctg1007	TC8563, TC27371^a^	3/44.7
Ctg636	TC31785*, TC27019^a^	na

^a^Markers that have not been genetically mapped

*Markers that are potentially involved in anthocyanin biosynthesis

**Table 6:** *Aquilegia *physical contigs mapped by 16 markers potentially involved in anthocyanin biosynthesis

TC Marker	Contig (length in kb)	Map position (Linkage group/cM)
TC32786	na	na
TC21211	ctg284 (3869)	na
TC21486	ctg768 (1190)	na
TC24814	ctg3509 (5544)	na
TC29760	ctg3160 (1671)	na
TC21880	ctg2888 (1403)	na
TC27360	ctg509 (1278)	1/12.9^a^
TC26594	ctg814 (1591)	na
TC21922	ctg1119 (2361)	na
TC33100	ctg823 (2190)	na
TC22565	ctg549 (2752)	na
TC30032	ctg348 (2228)	na
TC31785	ctg636 (668)	na
TC22820	ctg3306 (746)	na
TC26722	ctg537 (891)	na
TC30722	ctg849 (945)	na

^a^ctg509 was mapped based on a second marker, TC14816, in this contig

### Validation of the contig assembly

**Figure 2:**
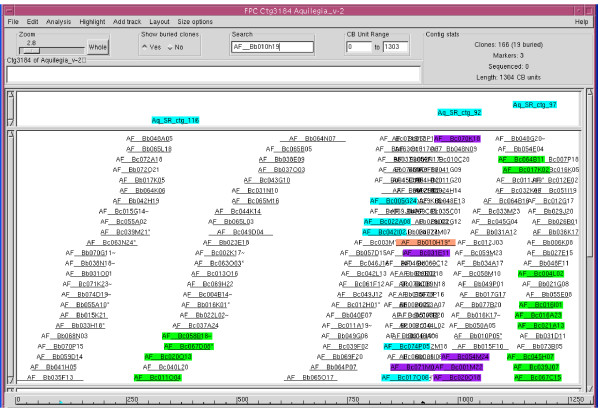
**Contig3184 of the *A. formosa *FPC build**. A total of 4 (labelled in green on the left), 6 (labelled in violet in the middle) and 9 (labelled in green on the right) BACs were positively hybridized to marker Aq_SR_ctg_116, Aq_SR_ctg_92 and Aq_SR_ctg_97, respectively. These BACs were neighbouring to each other in three clusters in association with corresponding markers. Hybridization of the BAC library with the probe derived from the BAC end sequence, AF_Bb010H19f (labelled in salmon orange) identified 8 BACs, five of which were also clustered in this contig as marked in light blue. Only AF_Bc library was used for the hybridization experiment.

**Figure 3:**
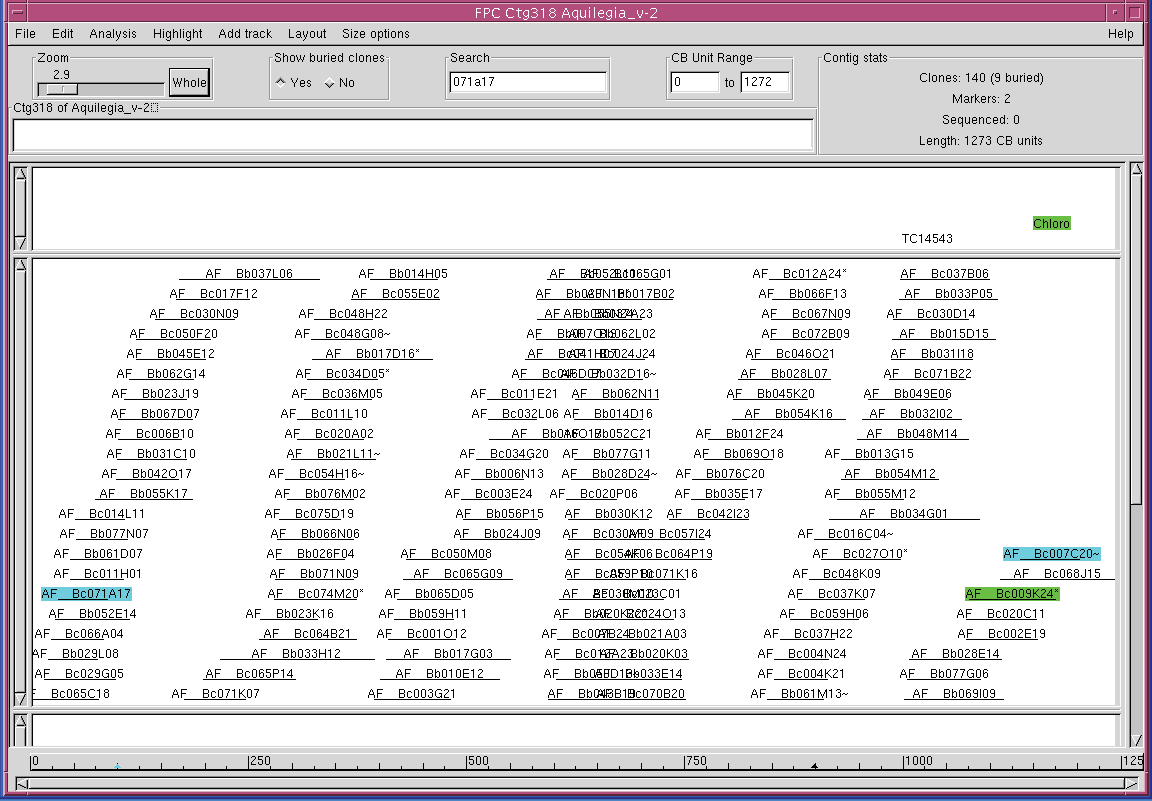
**Contig318 of the *A. formosa *FPC build**. Hybridization of the BAC library AF_Bc with the probe derived from the BAC end sequence, AF_Bb010H19f (labelled in salmon orange in Figure 2) also identified 2 clones at the ends of ctg318 (labelled in blue) possibly due to the presence of low copy homologous sequence shared with the probe.

Results from marker hybridization were also used to verify the contig assembly because, in theory, all clones that hybridized to a non-repetitive overgo probe are expected to be in the same contig. Alternatively, positive clones could be located at the ends of different contigs that do not have enough overlap to be assembled into a single contig under the stringency used for the analysis. On the other hand, BAC clones from unrelated contigs would be hybridized if an overgo probe happens to contain a repeat. To test these predictions we randomly chose ctg3184 (which contains 166 clones) from among those contigs that had multiple probe hybridizations. Pool hybridization anchored three markers to this contig. The first marker, Aq_SR_ctg_116, hybridized to 5 clones, among which were 4 clones (as labelled in green on the left of the contig in Figure 2) clustered in this contig, and the fifth clone was not included in the FPC project due to failed fingerprint. The second marker, Aq_SR_ctg_92, hybridized to 9 clones, of which 6 clones (Figure 2, labelled in violet) were neighbouring to each other in this contig, and of the remaining 3 clones 2 were singletons (AF__Bc070E01 and AF__Bc006O01) and 1 clone (AF__Bc044O24) did not fingerprint. The third marker, Aq_SR_ctg_97, resulted in 9 positive hits that were all clustering next to each other in this contig (Figure 2, green clones on the right). Furthermore, hybridization with the overgo probe derived from the T7-primed BAC end sequence of AF__Bb010H19f (Figure 2, labelled in salmon orange) from the same contig identified 8 clones, 5 of which were also next to each other in this contig (Figure 2, light blue-labelled clones). The remaining 3 BACs not in this contig were (a) 1 clone not included in the project due to the failed fingerprint and (b) 2 clones located at the very two ends of another contig, ctg318 (Figure 3, blue-labelled clones), possibly due to the presence of low copy homologous sequence shared with the probe. Thus, the hybridization results were consistent with and in support of the contig assembly, which is based on the fingerprint similarity of the BACs.

**Figure 4:**
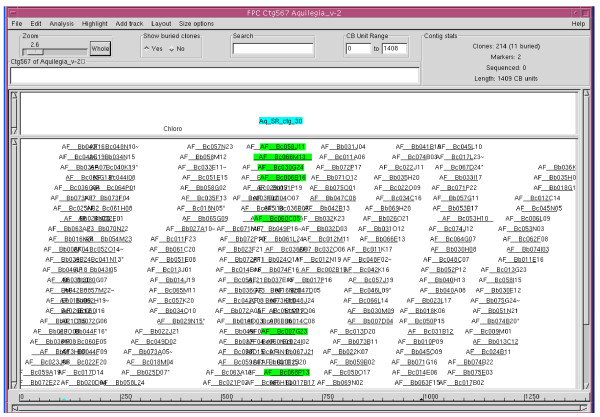
**Verification of FPC build**. Hybridization of the AF_BC BAC library with marker Aq_SR_ctg_30 (as highlighted in blue) identified 8 clones. Of these 8 clones were 7 clones (highlighted in green) in Ctg567 based on the FPC assembly. The remaining 1 clone did not have HICF data in the FPC and was therefore not seen in the build. All of these 8 clones generated amplicon of the expected size.

In another independent validation analysis, oligo primers were designed from a set of 8 markers for PCR amplification on all positively hybridized clones. As summarized in Table 7, other than Aq_SR_Ctg_8 and Aq_SR_Ctg_133, which only gave 10/12 and 3/4 successful PCR amplifications, respectively, the remaining 6 markers allowed amplification of expected amplicons from all positive clones. For example, overgo marker Aq_SR_Ctg_30 derived from an ubiquitin-conjugating enzyme E2 homolog hybridized to 8 positive BACs, and PCR analysis using a gene-specific primer pair generated matching amplicons from all these 8 positive clones, confirming the potential presence of the gene in these clones. The results were also consistent with the contig assembly in which 7 of the 8 clones were assembled in a patch in ctg567 (Figure 4, green-labeled clones) while the absence of the last BAC was due to failed HICF fingerprinting of the clone, which in turn excluded the clone from the framework physical map. In summary, a physical map was constructed through 3 major steps. First, an initial build was generated under high stringency with a Sulston score at 0.879. Second, contigs were further connected through a series of automated merges utilizing consecutive stepping-down stringencies from 1e-50 to 1e-35. Third, the physical map was further analyzed by manual editing of the contigs at lower cutoff at 1e-20 and tolerance at 3 according to marker hybridization data and an increased requirement for three end clone matches. The fidelity of the contig build could be confirmed by two sequence-based approaches, including (a) identification of neighbouring clones by hybridization with probes derived from BAC-end sequence of the same contig, and (b) analysis of the PCR amplicons generated from positively hybridized clones.

**Table 7:** Verification of FPC contig assembly by PCR amplicons designed hybridization markers.

Marker	Homology	Primer sequences	# positive hits (# contigs^a^)	# positive hits with expected amplicons
Aq_SR_Ctg_2	mlp-like protein 28	Fwd AGGTGATGGAACCTGTGAGG Rev CACAATCCATGTCACCAAGC	14 (3 +3s^b^)	14
Aq_SR_Ctg_8	No homology	Fwd GGCTATATCCACCAGGCTGA Rev AAGGGCCAGCACTTTATCCT	12 (3)	10
Aq_SR_Ctg_22	Late embryogenesis-abundant protein	Fwd ATCATCCAACCTTGCGTTGT Rev GGGACCGGAACTATCCAAAT	12 (2)	12
Aq_SR_Ctg_30	Ubiquitin-conjugating enzyme E2	Fwd GCCCAAATCAAGAAACCAGA Rev CCTTTATGGACCCTGGATCA	8 (1)	8
Aq_SR_Ctg_118	putative staygreen protein	Fwd TGGGGTCCACTTAAAGATGC Rev GAGTTGGTTGGTTTGGTTCC	5 (3)	5
Aq_SR_Ctg_127	universal stress protein 1	Fwd AGTAACTGGGCAAGCAGCAT Rev ATGGTGATGCAAGGGAAAAA	6 (2)	6
Aq_SR_Ctg_133	ethylene-responsive transcriptional co-activator)	Fwd ATCGCATCGTCATCAAACAA Rev TTCAGCAGGCGTACGACGAG	4 (2)	3
Aq_SR_Ctg_144	Benzodiazepine receptor-related	Fwd ACACTACGACATGCCAACCA Rev TAGCCCAGCCCAACAAATAG	2 (1)	2

^a^The number of contigs where the positively hybridized BACs were located

^b^Marker Aq_SR_Ctg_2 also hybridized to 3 singletons other than 11 clones in 3 different contigs

### BAC end sequencing

BAC end sequencing from both forward and reverse directions of 6,505 BACs covering a minimal tiling path of the physical framework generated a total of 12,086 (93% success) high quality sequences (at least 100 contiguous bases ≥ phred20) with an average length of 567 bases. This was equivalent to one sequence tag per 24.8 kb (considering the genome size of 300 Mb). After filtering for vector contamination and trimming for quality, the BESs were deposited in GenBank's GSS sequence repository: library AF_Bb has accessions ER936645-ER942217 and library AF_Bc has accessions ER967023-ER973759. Comparison of the BESs to multiple plant chloroplast and mitochondrial genomes indicated a low level of plastid-origin BACs, 0.2% for each organelle. The BAC sequences, excluding those of putative plastid origins, encompass 6,834,517 base pairs, which corresponds to approximately 2.3% of the *Aquilegia *genome [23].

The BAC end sequences have an average GC content of 37.6%. Microsatellites were identified from 2,091 BESs, and primers could be designed to flank 1,630 of the SSRs. These putatively mappable markers include 570 dinucleotide repeats, 550 trinucleotide repeats, 525 tetranucleotide repeats, and 177 pentanucleotide repeats.

A set of 1,729 sequences matched known transposable elements. Considering only the best matching element for each BAC end sequence, the most commonly matched species from the database was grapevine (*V. vinifera*) with 414 BAC ends having matches to grapevine elements. The matches encompassed 66 different repetitive elements including multiple members of the gypsy, copia, MuDR and En/Spm classes. These matches had an average Smith-Waterman score of 682. The next most common organisms for matches consisted of *A. thaliana *(370 BAC ends to 76 elements, score of 662), *Populus trichocarpa *(296 BAC ends to 43 elements, score of 878), followed by *Medicago truncatula *(166 BAC ends to 59 elements, score of 577), and *Oryza sativa *(143 BAC ends to 57 elements, score of 430).

The most commonly identified individual element was *Atlantys1_1 *that matched 143 *A. formosa *BAC ends. *Atlantys1_1 *represents an internal coding segment of the larger *Atlantys *endogenous retrovirus in the Ty3-*gypsy *family. This family is widespread across plants; *Atlantys *accounts for much of the genome size variation in rice [37] and also has RepBase records originating from *A. thaliana*, *Lotus japonicus*, and *Sorghum bicolor*. Other commonly identified elements include copia42-PTR_I, an LTR retrotransposon from *Populus *matching 83 BAC ends; POPGY1_I, the internal portion of a Gypsy-type retroelement matching 64 BAC ends; and Copia-31-lTR_VV, a LTR retrotransposon from *V. vinifera *matching 60 BAC ends (Additional file 1)

After filtration of organelle, transposable elements and repetitive sequences, the remaining BESs were assembled with CAP3 [38] followed by mining for potential gene coding regions. The assembly resulted in 8,140 singlets and 458 contigs. Two different strategies were used to identify potential coding regions in the unique sequences. In the first approach, the non-redundant dataset was compared to the tentative *Aquilegia *consensus EST sequences from the Gene Index Project [39] with tblastx [40] at a stringency of 1e-25. The results indicated 2,488 of the genomic sequences have at least one EST match. As the EST resource represented only an imperfect representation of the transcriptome, a BLASTX of *Arabidopsis*, *Oryza *and *Vitis *gene models was further performed with a cut-off E value of 1e-25 in the second approach, resulting in an additional 750, 337 and 921 potential coding non-redundant BAC ends, respectively. Of the 8598 non-redundant sequences, 2,782 (23% of the total 12,086 BESs) were flagged as potential coding regions.

**Figure 5:**
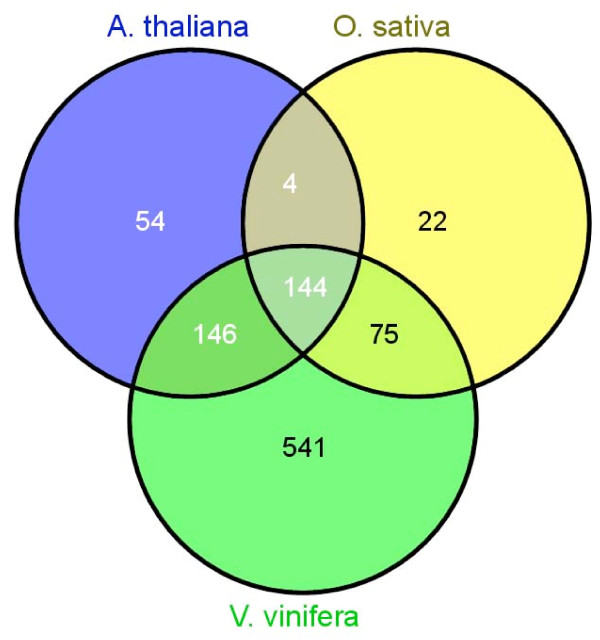
**Results of homology comparison (Venn diagram) of the *Aquilegia *BESs with 3 model genomes: *A. thaliana*, *O. sativa*, and *V. vinifera***. All BESs were filtered out repeats and transposable elements and compared against the model genomes by blastn search at 1e-10.

**Figure 6:**
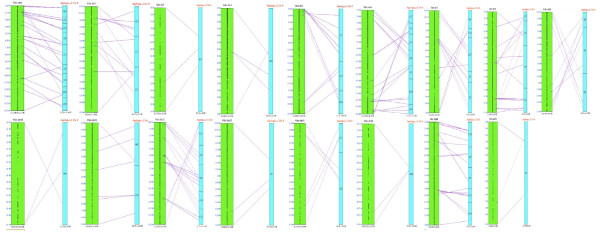
**Syntenies shared between *Aquilegia *and *V. vinifera***. A total of 54 syntenies, where both pair-end sequences from the same BAC match the same locus in *Vitis *genome, were identified by SyMAP analysis. The black dots in green *Vitis *bars indicate all annotated ESTs from the *Vitis *genome. The blue *Aquilegia *bars are the *Aquilegia *contigs that have syntenies aligned to *Vitis *genome. The name of each contig was described in each bar. The contigs were arranged based on the order of their corresponding orthologs in *Vitis *genome. The contigs assigned to the same *Aquilegia *"pseudochromosome" (marked as *Aquilegia *chr 0) may not overlap each other. There might be gaps among the contigs in the same "pseudochromosome". The purple lines connecting the green *Vitis *bars and blue *Aquilegia *bars indicate the match syntenies.

An overall comparison to three plant model genomes was performed by a blastn [40] of all the BESs to the whole genome sequences with an E-value cut-off of 1e-10. *Aquilegia *sequences exhibited relatively low similarity to *A. thaliana *and *O. sativa *with only 348 and 245 matches, respectively, while there were 906 matches with *V. vinifera *genome (Figure 5). The results provided the first global sequence information to support the phylogenetic placement of *Aquilegia *in angiosperms, and the observation from the shared transposable element between *Aquilegia *and the grapevine further reiterates the close colineage between these two clades. The fact that *Aquilegia *genome contains transposable elements similar to both monocot and eudicot species also highlights the uniqueness of *Aquilegia *in studying plant evolution. We identified 906 *Aquilegia *BAC-end sequences that aligned with one or more of the 19 chromosome-based pseudomolecules of the *Vitis *genome (Additional file 2) and 207 sequences aligned to unanchored *Vitis *genomic sequence. The alignment of the 906 *Aquilegia *BESs to the corresponding chromosome-based pseudomolecules of *Vitis *genome was summarized in Table 8. Using the series of synteny mapping algorithms, we mapped 54 blocks of synteny to the *V. vinifera *draft genome assembly (Figure 6).

**Table 8:** Comparative mapping of *Aquilegia *and *V. vinifera*^a^

Number of *Aquilegia *BESs	*Vitis *chromosome
70	1
62	2
33	3
44	4
81	5
100	6
48	7
69	8
28	9
23	10
45	11
36	12
37	13
72	14
23	15
25	16
40	17
79	18
45	19

^a^A summary of the alignment of the *Aquilegia *BESs to the corresponding chromosome-based pseudomolecules of *Vitis *genome.

## Discussion

*Aquilegia *represents a unique clade of basal eudicots possessing a number of important unique features, including its phylogenetic position in the lower eudicots, unusual floral morphology (e.g., petaloid sepals, nectar spurs and staminodia), and its distribution in diverse ecological habitats. Collectively, all these traits contributed to *Aquilegia *being developed as a new model system for studying floral variation, adaptive radiations and evolution [23,32,36]. To further understand the genome structure and provide molecular insights bridging monocots and eudicots and facilitate molecular dissection of the traits associated with inflorescence development and environmental adaptations, a BAC-based genomic resource, including three BAC libraries and a physical map, was developed in this study. Among the three libraries were two libraries derived from *A. formosa*, representing 15.2X and 13.3X genome equivalents, respectively, for physical map construction. A third library was constructed from *A. coerulea *Goldsmith to have 20.7X genome coverage for further comparative genomics studies to address the molecular basis for floral variation and adaptive radiation within the genus. The *Aquilegia *physical map was composed of 50,155 clones and had a deep 21X genome coverage. Furthermore, a collection of BACs orchestrating a minimal tiling path from the contig assembly were isolated for BAC end sequencing to provide a glimpse of the genome organization of this model plant. Both the physical map and the BESs could also serve as landmarks for genome sequence assembly and anchoring ESTs to the genome. Marker hybridizations using a total of 197 markers associated with drought-stress, anthocyanin biosynthesis and floral development not only allowed integration of genetic map into the contig framework, but also identified candidate genomic regions for further gene isolation and characterization. The genome resource is expected to serve as a pivotal platform for comparative genomics study to elucidate genome variations between monocots and basal eudicot and to provide insights into the molecular mechanisms underlying environment adaptation and floral variations.

In recent years, HICF fingerprinting has been commonly applied to replace traditional agarose [41] and polyacrylamide gel methods [42] in various genome fingerprinting projects due to its high-throughput procedure, increased number of fragments generated from each clone and more improved contig assembly than other approaches [43]. In this study, an average of 81 restriction fragments was generated from the clones in the FPC project. The high-informative fingerprints provided high resolution identity from each clone for accurate contig assembly that can be further verified by marker hybridization in which 189 (96%) of the total 197 genetic markers hybridized to only 1 or 2 contigs instead of scattering around the entire genome. Furthermore, the positively hybridized clones were overlapped in clusters in most contigs, indicating that the contig assembly, which is based on fingerprinting similarity, is consistent with the sequence-based results. The accuracy of contig assembly could also be verified by PCR amplicon analysis as shown in Table 7. Thus, we are confident with the strategy for building a physical map that begins with contig assembly at high stringency at cutoff 1e-50 and tolerance 3, which gave a high average Sulston score of 0.879, followed by a series of End-End and Single-End merges of the small BAC scaffolds under gradually decreased stringency till 1e-35, followed by further manual editing at 1e-20 based on marker hybridization data. Among the successful 197 markers used for the hybridization were 87 markers that have been genetically mapped; these markers anchored a total of 54 contigs that cover 76.4 Mb (25.5% of the genome) on all 7 linkage groups (Table 4). These mapped contigs not only organize a framework to study the *Aquilegia *genome, but also pave the way for gene isolation and characterization by map-based cloning approach to further understand the genes of interest.

The genes involved in anthocyanin pigmentation biosynthesis in wheat are arranged in a gene cluster in the short arm of chromosome 7 [44-46]. Similar clustering of the genes involved in the biosynthesis of secondary metabolites was also reported from grapevine [47]. Unlike these species, the 16 anthocyanin biosynthesis related genes in *Aquilegia *appear to be dispersed in the genome (Table 6), suggesting the unique deployment of the genes in this lower eudicot genus. However, a number of additional genes belonging to the anthocyanin and broader flavonoid pathway have been identified [36] but not assayed here, and therefore the possibility cannot be ruled out that some gene clustering might be identified in the future. The contigs anchored from this study could serve as resource for unravelling the molecular basis underlying floral color variation and evolution.

An expansion in the physical span of the contigs was observed in this study. The collective physical span of all contigs as calculated by the CB map function of FPC software [35] was estimated to be 689.8 Mb (~ 2.3X genome size, 1N = 300 Mb). As only 197 marker hybridization results were analyzed and these markers were biased toward specific biological functions, it cannot be ruled out, although unlikely, that the contig assembly is not best optimized and some contigs remain to be further merged together. As the single *A. formosa *individual used for BAC library construction has been shown to be highly heterozygous at more than 30 SSR and SNP loci (Hodges, unpublished data), the excessive physical length might be due to the heterozygous genome collected from the field that was composed of highly diverse haplotype DNAs as a result of the outcrossing nature of the species. Similar inflated length from physical map has been reported from other outcrossing species, including poplar [48] and grapevine [49]. As the genome sequencing project is near finishing, further assembly and analysis of genome sequence will uncover more details about the genome components and suggest events that took place affecting genome structure of this basal eudicot taxa. To maintain the accuracy in contig assembly, further reduction in stringency to merge more contigs was not pursued in this study. In the future, fingerprint contig assembly can be refined through more hybridizations using additional mapped markers and probes designed from the end clones of contigs.

The BESs from the minimal tiling path clones also provided insights into the genome composition of this novel model plant, including low GC content, transposable elements and gene content. Interestingly, higher homology in putative coding regions shared between *Aquilegia *and the grapevine, *V. vinifera*, in comparison to two other model plants, including rice and *Arabidopsis *was also observed (Figure 5). As *Vitis *is affiliated with the earliest diverging lineage of rosids in the core eudicots of the angiosperms [50], and *Aquilegia *is in basal eudicots in the phylogenetic tree [23,32], the close conservation between these two species not only provides a global molecular evidence to support the phylogenetic lineage that connects basal eudicots to core eudicots but also provides a rich resource for investigating the genome evolution, such as the events of genome duplication and subsequence variation [51-53], in the course from monocots to eudicots in angiosperms. In this report, preliminary comparative genomics studies using SyMAP uncovered 54 syntenic blocks between *Aquilegia *and *Vitis *(Figure 6). These syntenies provide a first glimpse of the *Aquilegia *structural organization and a rich resource to trace the events of DNA translocation during the evolution of these two lineages. Further characterization of the shared transposable elements from the *Aquilegia *genome will also provide insights into the evolution of plants. More extensive survey using the whole-genome sequence information in the near future is expected to aid in-depth studies into the evolution genomics of the basal eudicot taxa. On the other hand, the discovery that alignment of the BESs from the physical framework contigs failed to identify significant synteny with other reported genomes also reiterates the significance of the unique genome structure of *Aquilegia *in understanding the evolution of the plant genomes.

## Conclusions

The BAC-based genome resource established from this study, including deep genome coverage libraries from *A. formosa *and *A. coerulea*, a partially integrated physical map is expected to promote better understanding of the genome structure of the unique intermediate between rice and *Arabidopsis*. It will also provide tremendous insights into the molecular clues and genetic networks underlying ecological adaption and morphological diversity. Results from the analysis of the BESs derived from the minimal tiling path (MTP) indicated a close similarity in both transposable elements and annotated gene models with the grapevine genome further suggesting the significance of the genome resource in studying the molecular elements involved in the lineage of evolution progression. This genomic resource is expected to facilitate comparative genomics research, gene isolation and characterization to address the unique biological feature of this novel model plant.

## Methods

### BAC DNA fingerprinting and contig assembly

DNA was isolated from a total of 58,368 clones from both AF_Bb and AF_Bc BAC libraries by following standard alkaline lysis miniprep methods [54], and used for fingerprinting using the HICF method of Luo *et al. *[30]. The fingerprinting profiles were further processed by GeneMapper 3.7 (Applied Biosystems), GenoProfiler 2.0 [34], and uploaded to FPC v8.5.3 software [35] for contig assembly. To maintain the quality of contig assembly the initial build was processed at high stringency using the cutoff of 1e-50 and a tolerance of 3. The DQer function of the FPC package was performed to break down all contigs with more than 10% of Q clones to reduce false assembly. Further reassembly was conducted by consecutive reductions of the stringency at 1e-5 for the Ends-Ends analysis followed by Single-End analysis until the final cutoff of 1e-35 with tolerance of 3 was reached. The accuracy of the contig assembly was examined by marker hybridization and PCR analysis. Further manual editing of the assembly was conducted based on the following principles: (a) cutoff at 1e-20 and tolerance at 3, (b) for 2 contigs to be merged, the first contig needs to have at least 3 matched clones (matched clones are clones shared at least 41 common bands under the designated stringency) and the second contig needs to have at least 2 matched clones, (c) only 2 matched clones are required for contig merge if these 2 contigs also share the same genetic marker(s),

### Overgo design and hybridization

To establish a genome resource from an environmental and ecological model plant to better support gene identification and characterization, a collection of stress-induced genes were first chosen for hybridization to anchor the potential stress-related markers in the physical map. Furthermore, a BAC library from *A. coerulea *was also included in the hybridization for comparative genomics studies. Briefly, ESTs preferentially up-regulated by drought-stress were generated from subtractive hybridization analysis (Henry, unpublished data). The low complex sequences were further removed by a pipeline composed of Repeat Masker [55] with the RepBase database [56], Cross_Match [57] and Tandem Repeat Finder [58]. The remaining sequences were screened for overgo oligomers by OligoSpawn [59]. A total of 125 pairs of oligomers were synthesized by IDT (Integrated DNA Technologies). Overgo probes were individually labelled by following the procedure of the Clemson University Genomics Institute (CUGI) hybridization protocol http://www.genome.clemson.edu/resources/protocols. An in-house experimental design script http://www.genome.clemson.edu/software/hybdecon/exp_setup was used to assign probes into 15 pools in a 3-dimensional pooling design, with each pool containing 25 probes (Figure 1). All ^32^-P labelled probes were mixed in their corresponding pools, denatured and added to hybridization against 2 BAC libraries, including the AF_Bb library of *A. formosa *and a *Hind*III library of *A. coerulea*. Hybridization was performed at 60°C for 2 nights. Filters were washed with 1× SSC, 0.1% SDS at 60°C for 30 minutes for 5 times and exposed to phosphor screens, and the images were recorded by a Typhoon 9400 Imager (GE Healthcare, Bio-Sciences). The addresses of the positively hybridized BAC clones were manually scored using the software HybSweeper [60], and subsequently deconvoluted for positive BACs corresponding to each probe with an in-house PERL script Hybdecon http://www.genome.clemson.edu/software/hybdecon. Hybridization results were then incorporated into FPC project to anchor markers into the contig framework.

By following the same procedure, another set of 125 overgo probes was designed from various resources, including 87 mapped markers, 16 genes potentially involved in anthocyanin biosynthesis [36], 12 genes involved in floral development (Kramer, unpublished data) and 10 other SNP markers for additional pool hybridization. Successful markers were integrated into the map. Sequence information of all overgo probes were listed in Additional file 3.

### Contig validation by marker hybridization and PCR analysis

For PCR validation, primers were designed from a total of 8 markers randomly chosen from the drought-stress induced ESTs (Table 5). All positively hybridized BACs corresponding to every individual marker were analyzed by PCR amplification. The condition for the PCR reaction was 94°C for 1 min for initial denaturation, followed by 25 cycles of denaturing at 94°C for 15 sec, annealing at 55°C for 30 sec, and extension at 60°C for 60 sec, followed by a final cycle of extension for 10 min. The reagents were PCR kit from Clonetech (Palo Alto, CA). The amplicons were resolved in 1.0% agarose gel and ethidium bromide stained, and the presence/absence of the amplicons of expected sizes were examined.

### BAC end sequencing

A total of 6,505 overlapping BAC clones that constituted the minimal tiling path were rearrayed and cultured in 96-well deep plates for DNA isolation, and approximately 300 ng of each individual DNA was used for BAC end sequencing by universal T7 and Sp6 primers for both ends using the "Dye Terminator" chemistry from ABI kit version v3.1 and resolved on ABI3730XL sequencer. In-house quality control software was used to filter and trim raw sequences. The pipeline includes publicly available tools such as Phred [57], Cross_Match [57] and Lucy [61] for base calling and vector masking. Trimmed sequences of less than 100 bp or with greater than 5% N bases were removed. The high quality, trimmed sequences were searched for organelle origin by BLAST [40] against multiple genomes from GenBank: *A. thaliana*, *Nicotiana sylvestris*, *O. sativa*, and *Ranunculus macranthus *chloroplast genomes and the *A. thaliana*, *N. tobacum*, *O. sativa *and *V. vinifera *mitochondrial genomes. The software RepeatMasker version 3.2.7 [55] coupling with a RepBase library [56] of all known Viridiplantae repetitive elements was used to identify repeats from the *Aquilegia *BESs. Classification of the repeat families was based on the annotation in the database. A CUGI PERL script was used to identify microsatellites with at least five dinucleotide, four trinucleotide, three tetranucleotide or three pentanucleotide motifs in a row. Primer3 [62] was used to identify primers surrounding each predicted SSR element.

### Synteny mapping

BAC-end sequences anchored to fingerprint contigs were assessed for synteny with the *V. vinifera *draft genome assembly http://www.plantgdb.org/VvGDB using the SyMAP [63] software. First, repetitive/low-complex motifs were screened and masked with Repeatmasker [55]. Next, BLAT [64] was used to align the FPC sequences (BES and markers) using the nucleotide/nucleotide search mode with a minScore of 30 and a minIdentity of 70.

## Authors' contributions

GF and BPB contributed equally to the major part of the study. DCH performed subtractive hybridization for drought-stress marker identification, BAC end sequencing and contig validation. MES performed all bioinformatic analysis. CAS helped with SyMAP analysis and discussion. SAH provided the *A. formosa *libraries and the genetically mapped and anthocyanin biosynthesis marker information. JPT and HL coordinated the project and HL drafted the manuscript. All authors read and approved the final draft of the manuscript.

## Supplementary Material

Additional file 1 **The top 16 most common repetitive elements in *A. formosa *BESs identified**. The transposable elements were identified from *Aquilegia *BESs using RepeatMasker coupling with a RepBase library of all known Viridiplantae repetitive elements. The elements were listed according to the number of reads of each element in a descending order as described in column 4. Click here for file

Additional file 2 **Identification of syntenies between *A. formosa *and *V. vinifera genomes***. BESs were compared with *V. vinifera *genome using the cutoff at 1e-10 and the matches were listed in the table. *Aquilegia *framework contig and BAC were listed in column 1, the number of BACs in the corresponding contig was listed in column 2, putative gene function of the annotated *Vitis *ortholog was described in column 3, and linkage group where the *Vitis *ortholog is located was described in column 4. Click here for file

Additional file 3 **Spread sheet of the detail sequence information of the overgo probes used in this study**. The probes with the nomenclature of Aq_SR_ctg and AHOTEg were derived from drought stress ESTs, while the TC probes were from a list of genes potentially involved in anthocyanin biosynthesis or floral development. To generate the probes, marker sequences were processed through a pipeline composed of RepeatMasker, Cross-Match and Tandem Repeat Finder to remove low complex sequence regions before screening for overgo oligomers by OligoSpawn. Click here for file
