# Supplementary material for: Genomic tools development for Aquilegia: construction of a BAC-based physical map
Source: BMC Genomics. 2010 Nov 8;11:621. doi: 10.1186/1471-2164-11-621 (PMC3091760; doi:10.1186/1471-2164-11-621)
Supplement: Additional file 1 — The top 16 most common repetitive elements in A. formosa BESs identified. The transposable elements were identified from Aquilegia BESs using RepeatMasker coupling with a RepBase library of all known Viridiplantae repetitive elements. The elements were listed according to the number of reads of each element in a descending order as described in column 4. [file 1471-2164-11-621-S1.DOC]

**Additional file 1: The top 16 most common repetitive elements in *A. formosa*** BESs identified by RepeatMasker

| RepBase element | RepBase description | RepBase organism source | # *Aquilegia BESs* |
| --- | --- | --- | --- |
| ATLANTYS1_I | An internal portion of the ATLANTYS1 endogenous retrovirus | *Arabidopsis thaliana* | 143 |
| Copia42-PTR_I | LTR retrotransposon from Populus tricho-carpa: internal portion | *Populus trichocarpa* | 83 |
| POPGY1_I | Gypsy-type retroelement, internal portion | *Populus trichocarpa* | 64 |
| Copia-31-LTR_VV | Copia-31_VV, LTR retrotransposon Ty1-copia like, internal portion | *Vitis vinifera* | 60 |
| Gypsy16-VV_I | LTR retrotransposon from grapevine: internal portion | *Vitis vinifera* | 47 |
| LSU-rRNA_Ath | Long Subunit rRNA pseudogene | General to *Viridiplantae* | 47 |
| SSU-rRNA_Ath | Short Subunit rRNA Pseudogene | General to *Viridiplantae* | 39 |
| SHACOP17_I_MT | Internal region of LTR retroposon, SHACOP17_MT | *Medicago truncatula* | 29 |
| ATCOPIN_LTR | Non-autonomous LTR retrotransposon – consensus LTR | *Arabidopsis thaliana* | 27 |
| Gypsy3-VV_ILTR | Retrotransposon from grapevine: internal portion | *Vitis vinifera* | 25 |
| Copia16-VV_I LTR | Retrotransposon from grapevine: internal portion | *Vitis vinifera* | 23 |
| GYPSI_I | Gypsy-like element | *Oryza sativa* | 23 |
| Gypsy18-VV_I | LTR retrotransposon from grapevine: internal portion | *Vitis vinifera* | 23 |
| ATCOPIA43I | Internal portion of the ATCOPIA43 copia-like  endogenous retrovirus –  a consensus sequence | *Arabidopsis thaliana* | 20 |
| ATLANTYS2_I | ATLANTYS2_I is an internal portion of the ATLANTYS2 endogenous retrovirus - a consensus sequence | *Arabidopsis thaliana* | 20 |
| Gypsy1-PTR_I | LTR retrotransposon, internal portion | *Populus trichocarpa* | 20 |
