# Supplementary material for: Genomic tools development for Aquilegia: construction of a BAC-based physical map
Source: BMC Genomics. 2010 Nov 8;11:621. doi: 10.1186/1471-2164-11-621 (PMC3091760; doi:10.1186/1471-2164-11-621)
Supplement: Additional file 2 — Identification of syntenies between A. formosa and V. vinifera genomes. BESs were compared with V. vinifera genome using the cutoff at 1e-10 and the matches were listed in the table. Aquilegia framework contig and BAC were listed in column 1, the number of BACs in the corresponding contig was listed in column 2, putative gene function of the annotated Vitis ortholog was described in column 3, and linkage group where the Vitis ortholog is located was described in column 4. [file 1471-2164-11-621-S2.DOC]

Additional file 2: Syntenic conservation between *A. formosa* and *V. vinifera genomes.*

| Aquilegia sequence/contig | No. Clones | *Vitis* Ortholog | *Vitis* chromosome |
| --- | --- | --- | --- |
| Ctg1026_AF__Bb0024D07r | 80 | ---NA--- | chr1 |
| Ctg1036_AF__Bb0055K02f | 30 | spotted leaf | chr1 |
| Ctg1135_AF__Bb0026C23f | 9 | protein | chr1 |
| Ctg1159_AF__Bb0007H17f | 24 | rna-dependent rna polymerase | chr1 |
| Ctg1159_AF__Bc0051G05f | 24 | amino acid transporter | chr1 |
| Ctg1212_AF__Bb0036G05r | 25 | protein | chr1 |
| Ctg1221_AF__Bb0025C11f | 77 | endoxyloglucan transferase | chr1 |
| Ctg1290_AF__Bc0002J23r | 39 | peptide transporter-like protein | chr1 |
| Ctg1327_AF__Bb0010H09r | 58 | nac domain ipr003441 | chr1 |
| Ctg1353_AF__Bb0053G20r | 42 | protein | chr1 |
| Ctg1359_AF__Bc0029N04r | 42 | PREDICTED: hypothetical protein [Vitis vinifera] | chr1 |
| Ctg143_AF__Bc0004P14f | 75 | integral membrane protein | chr1 |
| Ctg1434_AF__Bb0066M16f | 75 | protein | chr1 |
| Ctg1452_AF__Bc0018A07f | 21 | at5g38200 mxa21_90 | chr1 |
| Ctg1517_AF__Bc0021B13r | 8 | protein | chr1 |
| Ctg1568_AF__Bb0031C21f | 79 | protein binding | chr1 |
| Ctg1568_AF__Bc0037J09f | 79 | receptor-kinase isolog | chr1 |
| Ctg1577_AF__Bb0077E06r | 79 | transparent testa 1 | chr1 |
| Ctg1577_AF__Bc0032G01f | 79 | protein kinase family protein | chr1 |
| Ctg1599_AF__Bb0069C14r | 131 | trehalose 6-phosphate synthase | chr1 |
| Ctg177_AF__Bc0056M07r | 34 | zinc finger | chr1 |
| Ctg216_AF__Bc0021J06r | 6 | xyloglucan endotransglucosylase hydrolase protein a | chr1 |
| Ctg2355_AF__Bb0043L13f | 41 | protein | chr1 |
| Ctg2355_AF__Bb0065B14r | 41 | protein | chr1 |
| Ctg2541_AF__Bb0024K11r | 57 | protein | chr1 |
| Ctg2541_AF__Bb0034M18f | 57 | pentatricopeptide repeat-containing | chr1 |
| Ctg2541_AF__Bc0063H06r | 57 | protein | chr1 |
| Ctg2571_AF__Bb0017D23r | 30 | increased size exclusion limit 2 | chr1 |
| Ctg258_AF__Bb0045K10r | 3 | importin 9 | chr1 |
| Ctg2602_AF__Bb0016F20r | 11 | phosphoenolpyruvate carboxylase | chr1 |
| Ctg286_AF__Bb0002I06r | 4 | phytosulfokine receptor | chr1 |
| Ctg313_AF__Bb0045J07f | 2 | amino acid permease | chr1 |
| Ctg4172_AF__Bb0050I05f | 32 | protein | chr1 |
| Ctg418_AF__Bc0001J19f | 26 | PREDICTED: hypothetical protein [Vitis vinifera] | chr1 |
| Ctg4216_AF__Bb0040B23f | 11 | pentatricopeptide repeat-containing | chr1 |
| Ctg4661_AF__Bb0044O10f | 3 | oligopeptide transporter opt family | chr1 |
| Ctg537_AF__Bb0042K03r | 17 | starch phosphorylase | chr1 |
| Ctg600_AF__Bc0028I24f | 17 | glycyl-trna synthetase | chr1 |
| Ctg644_AF__Bc0024D14f | 17 | atp-dependent rna | chr1 |
| Ctg646_AF__Bb0023J21r | 17 | ran gtpase binding | chr1 |
| Ctg683_AF__Bb0011F19f | 17 | protein | chr1 |
| Ctg683_AF__Bb0062N06r | 17 | protein | chr1 |
| Ctg697_AF__Bb0046H07f | 17 | transport protein | chr1 |
| Ctg791_AF__Bb0039K07f | 17 | protein | chr1 |
| Ctg791_AF__Bb0039K07r | 17 | magnesium transporter | chr1 |
| Ctg792_AF__Bb0011P17f | 17 | ---NA--- | chr1 |
| Ctg81_AF__Bb0012A24f | 17 | respiratory burst | chr1 |
| Ctg921_AF__Bc0062L05r | 17 | ---NA--- | chr1 |
| Ctg937_AF__Bb0015E15r | 17 | immediate-early fungal elicitor protein cmpg1 | chr1 |
| Ctg977_AF__Bb0016D18r | 17 | protein | chr1 |
| Ctg977_AF__Bc0061A05f | 17 | dna binding | chr1 |
| Ctg979_AF__Bc0035K15r | 17 | PREDICTED: hypothetical protein [Vitis vinifera] | chr1 |
| Ctg1056_AF__Bb0072J16r | 34 | unnamed protein product [Vitis vinifera] | chr1_random |
| Ctg1077_AF__Bb0030B01f | 25 | e3 ubiquitin ligase | chr1_random |
| Ctg1268_AF__Bb0068B14r | 16 | phosphatidylinositol 3-kinase | chr1_random |
| Ctg1393_AF__Bc0003G24f | 66 | protein | chr1_random |
| Ctg1509_AF__Bb0069P04f | 13 | brassinosteroid insensitive 1-associated receptor kinase 1 | chr1_random |
| Ctg192_AF__Bb0004P17r | 3 | gag-pol identical | chr1_random |
| Ctg283_AF__Bb0015L19f | 2 | alpha-glucan water chloroplast | chr1_random |
| Ctg283_AF__Bc0060C12r | 2 | protein | chr1_random |
| Ctg363_AF__Bb0022M20r | 4 | mitochondrial chaperone | chr1_random |
| Ctg3796_AF__Bb0021G07r | 42 | histidine kinase cytokinin receptor | chr1_random |
| Ctg385_AF__Bb0017J12r | 6 | peptidase family-like protein | chr1_random |
| Ctg3960_AF__Bc0055J17f | 7 | nucleic acid binding | chr1_random |
| Ctg420_AF__Bc0004B11f | 33 | lysine histidine transporter | chr1_random |
| Ctg4684_AF__Bc0025L14r | 64 | multidrug resistance protein abc transporter family | chr1_random |
| Ctg723_AF__Bb0069O08f | 17 | multidrug resistance protein abc transporter family | chr1_random |
| Ctg870_AF__Bb0053O19r | 17 | signal recognition particle receptor beta subunit | chr1_random |
| Ctg951_AF__Bb0048C24r | 17 | signal recognition particle subunit | chr1_random |
| Ctg978_AF__Bb0013E21r | 17 | ---NA--- | chr1_random |
| Ctg1059_AF__Bb0055D24f | 28 | minichromosome maintenance protein | chr2 |
| Ctg1066_AF__Bb0051O02r | 10 | protein | chr2 |
| Ctg1081_AF__Bc0016B24r | 7 | ---NA--- | chr2 |
| Ctg1087_AF__Bb0022M24r | 22 | ---NA--- | chr2 |
| Ctg1101_AF__Bc0014N09f | 24 | retrotransposon unclassified | chr2 |
| Ctg1116_AF__Bc0029B10r | 47 | atp binding | chr2 |
| Ctg1116_AF__Bc0044M07r | 47 | cytochrome p450 | chr2 |
| Ctg1227_AF__Bb0064D08r | 27 | protein | chr2 |
| Ctg1263_AF__Bc0034E02r | 22 | Os07g0297300 [Oryza sativa Japonica Group] | chr2 |
| Ctg1318_AF__Bc0040B04f | 5 | protein | chr2 |
| Ctg1390_AF__Bc0066P11r | 20 | ap2 erf domain-containing transcription factor | chr2 |
| Ctg1688_AF__Bc0009B17f | 7 | unknown [Zea mays] | chr2 |
| Ctg1709_AF__Bb0056I22f | 9 | ---NA--- | chr2 |
| Ctg1753_AF__Bc0042L11r | 16 | cytochrome p450 like_tbp | chr2 |
| Ctg2_AF__Bc0001E23f | 10 | cytochrome p450 like_tbp | chr2 |
| Ctg2_AF__Bc0001E23r | 10 | protein | chr2 |
| Ctg2_AF__Bc0004C11f | 10 | hypothetical protein G11MC16DRAFT_3625 [Geobacillus sp. G11MC16] | chr2 |
| Ctg2_AF__Bc0004C11r | 10 | protein | chr2 |
| Ctg2_AF__Bc0014B23r | 10 | cytochrome p450 like_tbp | chr2 |
| Ctg2_AF__Bc0018G13f | 10 | cytochrome p450 like_tbp | chr2 |
| Ctg2_AF__Bc0028D12r | 10 | protein | chr2 |
| Ctg2_AF__Bc0052O08r | 10 | cytochrome p450 like_tbp | chr2 |
| Ctg2_AF__Bc0054M08f | 10 | 10 kda secreted protein | chr2 |
| Ctg2_AF__Bc0059P05f | 10 | cytochrome p450 like_tbp | chr2 |
| Ctg2_AF__Bc0060O17f | 10 | hypothetical protein Ssol98_08391 [Sulfolobus solfataricus 98/2] | chr2 |
| Ctg2_AF__Bc0072E04r | 10 | 10 kda secreted protein | chr2 |
| Ctg2_AF__Bc0073M04r | 10 | cytochrome p450 like_tbp | chr2 |
| Ctg2024_AF__Bc0040M08r | 173 | heat shock transcription factor 1 | chr2 |
| Ctg203_AF__Bb0073J17r | 3 | 2c-methyl-d-erythritol -cyclodiphosphate synthase | chr2 |
| Ctg2122_AF__Bc0014B04f | 7 | jumonji domain protein | chr2 |
| Ctg2122_AF__Bc0075E08r | 7 | leucine-rich repeat receptor-like protein kinase | chr2 |
| Ctg2546_AF__Bb0057E16f | 6 | protein | chr2 |
| Ctg261_AF__Bc0026F04f | 4 | rnase l inhibitor-like protein | chr2 |
| Ctg277_AF__Bc0044I22f | 3 | minichromosome maintenance protein | chr2 |
| Ctg288_AF__Bc0011A04f | 3 | gag-pol polyprotein | chr2 |
| Ctg3265_AF__Bc0041G08f | 128 | hypothetical protein Ssol98_08391 [Sulfolobus solfataricus 98/2] | chr2 |
| Ctg3265_AF__Bc0041I06r | 128 | 10 kda secreted protein | chr2 |
| Ctg3265_AF__Bc0071L10f | 128 | cytochrome p450 like_tbp | chr2 |
| Ctg3265_AF__Bc0071L10r | 128 | hypothetical protein G11MC16DRAFT_3625 [Geobacillus sp. G11MC16] | chr2 |
| Ctg3299_AF__Bc0047I06r | 22 | rrna promoter binding | chr2 |
| Ctg333_AF__Bb0057I15r | 3 | protein | chr2 |
| Ctg3527_AF__Bc0018C23f | 9 | gag-pol polyprotein | chr2 |
| Ctg356_AF__Bc0067G20r | 109 | gag-pol polyprotein | chr2 |
| Ctg3701_AF__Bc0040M19f | 13 | protein | chr2 |
| Ctg3786_AF__Bb0048N06f | 4 | unnamed protein product [Vitis vinifera] | chr2 |
| Ctg3818_AF__Bb0055H12f | 34 | gag-pol polyprotein | chr2 |
| Ctg4125_AF__Bc0039J03f | 43 | wnk7 kinase protein kinase | chr2 |
| Ctg4332_AF__Bc0033H07r | 33 | gag-pol polyprotein | chr2 |
| Ctg511_AF__Bc0008C17r | 17 | 10 kda secreted protein | chr2 |
| Ctg524_AF__Bc0017I23r | 17 | protein | chr2 |
| Ctg524_AF__Bc0056J24r | 17 | 10 kda secreted protein | chr2 |
| Ctg579_AF__Bb0066L20r | 17 | acyl- synthetase | chr2 |
| Ctg596_AF__Bb0044F03f | 17 | photomorphogenesis repressor protein | chr2 |
| Ctg635_AF__Bc0056L01r | 17 | gag-pol polyprotein | chr2 |
| Ctg636_AF__Bb0032G08r | 17 | xylem serine proteinase 1 | chr2 |
| Ctg66_AF__Bb0019H21r | 17 | protein | chr2 |
| Ctg666_AF__Bc0065G11r | 17 | nucleoside diphosphate kinase 3 | chr2 |
| Ctg723_AF__Bc0029G04f | 17 | pattern formation | chr2 |
| Ctg756_AF__Bc0040J08f | 17 | gag-pol polyprotein | chr2 |
| Ctg801_AF__Bc0067B11f | 17 | PREDICTED: similar to binding [Vitis vinifera] | chr2 |
| Ctg891_AF__Bc0070O18r | 17 | vacuolar protein sorting-associated | chr2 |
| Ctg957_AF__Bc0049B18f | 17 | nucleoporin family protein | chr2 |
| Ctg10_AF__Bb0017D14f | 80 | ---NA--- | chr3 |
| Ctg114_AF__Bc0026F08f | 38 | hypothetical protein [Vitis vinifera] | chr3 |
| Ctg1163_AF__Bb0030M07r | 15 | ---NA--- | chr3 |
| Ctg1219_AF__Bc0049A15r | 17 | dna binding | chr3 |
| Ctg1462_AF__Bb0034O02r | 8 | ---NA--- | chr3 |
| Ctg1648_AF__Bc0034E06f | 13 | glutathione-regulated potassium-efflux system protein | chr3 |
| Ctg2041_AF__Bb0013G08f | 5 | #NAME? | chr3 |
| Ctg249_AF__Bb0044J17r | 5 | isp4-like protein | chr3 |
| Ctg332_AF__Bb0044K03r | 3 | serine threonine-protein kinase | chr3 |
| Ctg347_AF__Bb0004H19r | 20 | ---NA--- | chr3 |
| Ctg363_AF__Bb0022M20f | 4 | saur family protein | chr3 |
| Ctg363_AF__Bb0023D16f | 4 | ---NA--- | chr3 |
| Ctg371_AF__Bb0066J12f | 2 | protein | chr3 |
| Ctg3817_AF__Bc0043A06f | 65 | phenylcoumaran benzylic ether reductase | chr3 |
| Ctg3891_AF__Bb0026H09f | 23 | phosphatidylinositol 4- | chr3 |
| Ctg4208_AF__Bc0063J07r | 50 | dj-1 family protein | chr3 |
| Ctg4208_AF__Bc0067O03f | 50 | nitrite reductase | chr3 |
| Ctg4249_AF__Bc0063M08f | 20 | calcium-binding ef hand family protein | chr3 |
| Ctg599_AF__Bc0041B11f | 17 | c-type cytochrome biogenesis protein | chr3 |
| Ctg654_AF__Bc0075A11r | 17 | ---NA--- | chr3 |
| Ctg814_AF__Bc0017E24f | 17 | snf2 super family | chr3 |
| Ctg91_AF__Bb0016C03r | 17 | exocyst complex component | chr3 |
| Ctg962_AF__Bc0044L04f | 17 | ---NA--- | chr3 |
| Ctg968_AF__Bb0064G14r | 17 | ---NA--- | chr3 |
| Ctg1536_AF__Bc0032D07r | 17 | deoxycytidyl transferase | chr3_random |
| Ctg18_AF__Bb0028G13f | 4 | polynucleotidyl ribonuclease h fold | chr3_random |
| Ctg1937_AF__Bb0076P05r | 7 | polynucleotidyl ribonuclease h fold | chr3_random |
| Ctg234_AF__Bb0060P18f | 4 | polynucleotidyl ribonuclease h fold | chr3_random |
| Ctg3565_AF__Bb0010P06f | 7 | gag-pol protein | chr3_random |
| Ctg381_AF__Bb0056F22r | 9 | retrotransposon ty3-gypsy subclass | chr3_random |
| Ctg3856_AF__Bb0035G15r | 42 | retrotransposon ty3-gypsy subclass | chr3_random |
| Ctg3891_AF__Bb0068N08f | 23 | hypothetical protein [Vitis vinifera] | chr3_random |
| Ctg92_AF__Bb0062D09r | 17 | polynucleotidyl ribonuclease h fold | chr3_random |
| Ctg10_AF__Bc0071J17f | 80 | ---NA--- | chr4 |
| Ctg1040_AF__Bb0033I18r | 8 | ---NA--- | chr4 |
| Ctg106_AF__Bb0010F09f | 9 | transcriptional corepressor leunig | chr4 |
| Ctg1071_AF__Bb0034A06f | 19 | nadp-dependent glyceraldehyde-3-phosphate dehydrogenase | chr4 |
| Ctg1078_AF__Bb0028L16f | 20 | vacuolar processing enzyme | chr4 |
| Ctg1097_AF__Bc0068B22r | 7 | ---NA--- | chr4 |
| Ctg113_AF__Bc0064O15r | 6 | nucleolysin tia- | chr4 |
| Ctg1228_AF__Bc0006L18r | 8 | ---NA--- | chr4 |
| Ctg1289_AF__Bb0056K22r | 15 | protein | chr4 |
| Ctg1597_AF__Bc0063L18f | 7 | hec3 (hecate 3) dna binding transcription factor | chr4 |
| Ctg168_AF__Bb0019C11r | 9 | eukaryotic initiation factor 5c cg2922- isoform f | chr4 |
| Ctg168_AF__Bb0023L12r | 9 | auxin efflux carrier component | chr4 |
| Ctg1700_AF__Bb0007B03f | 17 | hydroxyproline-rich glycoprotein family protein | chr4 |
| Ctg1753_AF__Bb0063D16f | 16 | serine-threonine protein plant- | chr4 |
| Ctg181_AF__Bc0029G14f | 5 | 26s proteasome regulatory particle triple-a atpase subunit6 | chr4 |
| Ctg193_AF__Bb0044A07f | 6 | glucose inhibited division protein a | chr4 |
| Ctg1947_AF__Bc0052C07r | 7 | transferring glycosyl | chr4 |
| Ctg2070_AF__Bc0057M09r | 7 | gag-pol polyprotein | chr4 |
| Ctg255_AF__Bc0008B06r | 2 | protein | chr4 |
| Ctg259_AF__Bc0069O11r | 4 | protein | chr4 |
| Ctg315_AF__Bb0032D14r | 2 | nadp-dependent glyceraldehyde-3-phosphate dehydrogenase | chr4 |
| Ctg3591_AF__Bc0041B09r | 64 | protein phosphatase regulatory | chr4 |
| Ctg363_AF__Bc0061D13r | 4 | saur family protein | chr4 |
| Ctg38_AF__Bb0048O15r | 30 | protein | chr4 |
| Ctg3817_AF__Bc0015N08r | 65 | integral membrane protein | chr4 |
| Ctg3873_AF__Bc0010K01r | 6 | puromycin-sensitive aminopeptidase | chr4 |
| Ctg3947_AF__Bc0010I21f | 4 | protein | chr4 |
| Ctg4339_AF__Bc0069J09f | 23 | chromatin remodeling complex subunit | chr4 |
| Ctg4385_AF__Bc0029A13r | 61 | retrotransposon ty3-gypsy sub-class | chr4 |
| Ctg4409_AF__Bc0025K09r | 43 | set domain protein sdg117 | chr4 |
| Ctg448_AF__Bc0062C06r | 9 | saur family protein | chr4 |
| Ctg486_AF__Bc0064A07f | 17 | ---NA--- | chr4 |
| Ctg507_AF__Bc0033E13r | 17 | hec3 (hecate 3) dna binding transcription factor | chr4 |
| Ctg512_AF__Bb0061A05r | 17 | chromatin remodeling complex subunit | chr4 |
| Ctg515_AF__Bb0029C14r | 17 | pentatricopeptide repeat-containing protein | chr4 |
| Ctg54_AF__Bc0050M07f | 17 | translational activator | chr4 |
| Ctg706_AF__Bc0071I24f | 17 | kinesin-like calmodulin-binding protein | chr4 |
| Ctg799_AF__Bc0074C07r | 17 | ferrochelatase precusor | chr4 |
| Ctg800_AF__Bb0004G11f | 17 | ---NA--- | chr4 |
| Ctg817_AF__Bc0021K13r | 17 | ---NA--- | chr4 |
| Ctg842_AF__Bb0029C17r | 17 | mitochondrial carrier protein | chr4 |
| Ctg912_AF__Bc0010N18r | 17 | lactoylglutathione glyoxalase | chr4 |
| Ctg94_AF__Bc0001I21r | 17 | udp-glucose pyrophosphorylase | chr4 |
| Ctg992_AF__Bb0074A22r | 17 | cytochrome p450 monooxygenase | chr4 |
| Ctg1_AF__Bc0066E11r | 30 | af478377_1gag-pol polyprotein | chr5 |
| Ctg1008_AF__Bb0069N03f | 16 | protein | chr5 |
| Ctg1029_AF__Bb0070P16f | 22 | extracellular signal-regulated kinase expressed | chr5 |
| Ctg1194_AF__Bc0035I02f | 16 | inosine-uridine preferring nucleoside | chr5 |
| Ctg1194_AF__Bc0070J10f | 16 | protein | chr5 |
| Ctg1232_AF__Bc0049I17r | 19 | unconventional myosin heavy chain | chr5 |
| Ctg1306_AF__Bb0068F05r | 31 | serine-threonine protein plant- | chr5 |
| Ctg1343_AF__Bb0028E09r | 15 | ---NA--- | chr5 |
| Ctg1350_AF__Bc0069G11f | 7 | protein | chr5 |
| Ctg140_AF__Bb0005M24r | 11 | hypothetical protein Poptr_cp075 [Populus trichocarpa] | chr5 |
| Ctg1454_AF__Bc0064O14f | 7 | est gb | chr5 |
| Ctg1474_AF__Bc0037C05r | 7 | isoleucyl trna | chr5 |
| Ctg1475_AF__Bc0021H23r | 17 | protein | chr5 |
| Ctg153_AF__Bb0032D20r | 10 | acyl- binding | chr5 |
| Ctg163_AF__Bb0006H04r | 5 | chr17 (chromatin remodeling factor17) atp binding dna binding dna-dependent atpase helicase acting on acid in phosphorus-containing anhydrides nucleic acid binding nucleosome binding | chr5 |
| Ctg163_AF__Bb0032N13r | 5 | syd atpase chromatin binding | chr5 |
| Ctg1642_AF__Bc0046F09f | 7 | protein | chr5 |
| Ctg1685_AF__Bb0005E13f | 28 | transport inhibitor response 1 | chr5 |
| Ctg1728_AF__Bc0043F08f | 10 | g1121 protein | chr5 |
| Ctg1775_AF__Bc0063B11r | 9 | multidrug pheromone mdr abc transporter family | chr5 |
| Ctg2_AF__Bc0041C17f | 10 | protein | chr5 |
| Ctg2_AF__Bc0044A01r | 10 | protein | chr5 |
| Ctg2_AF__Bc0071J22f | 10 | protein | chr5 |
| Ctg2_AF__Bc0073G09r | 10 | protein | chr5 |
| Ctg2049_AF__Bb0063N08f | 5 | protein | chr5 |
| Ctg2049_AF__Bb0063N08r | 5 | pyrimidine-specific ribonucleoside hydrolase riha | chr5 |
| Ctg2065_AF__Bb0070O12f | 86 | PREDICTED: hypothetical protein [Vitis vinifera] | chr5 |
| Ctg208_AF__Bc0063I01r | 5 | binding catalytic | chr5 |
| Ctg21_AF__Bb0007E01r | 41 | acting on acid in phosphorus-containing anhydrides nucleic acid binding zinc ion binding | chr5 |
| Ctg21_AF__Bb0041M12f | 41 | acting on acid in phosphorus-containing anhydrides nucleic acid binding zinc ion binding | chr5 |
| Ctg2142_AF__Bb0026N17f | 5 | retrotransposon ty1-copia subclass | chr5 |
| Ctg234_AF__Bc0006O17f | 4 | chromatin remodeling complex subunit | chr5 |
| Ctg2513_AF__Bb0053K21r | 4 | ---NA--- | chr5 |
| Ctg259_AF__Bc0064N02f | 4 | gag-protease-integrase-rt-r polyprotein | chr5 |
| Ctg274_AF__Bb0070P15r | 2 | multidrug pheromone mdr abc transporter family | chr5 |
| Ctg275_AF__Bc0039K10r | 2 | btb poz domain containing protein | chr5 |
| Ctg298_AF__Bb0013I20r | 2 | male sterility | chr5 |
| Ctg2992_AF__Bc0038A18r | 9 | protein | chr5 |
| Ctg302_AF__Bb0075P09r | 2 | boron transporter | chr5 |
| Ctg320_AF__Bc0070C21r | 5 | phospholipase d | chr5 |
| Ctg321_AF__Bb0070C06f | 5 | ---NA--- | chr5 |
| Ctg3265_AF__Bc0056D06r | 128 | protein | chr5 |
| Ctg3299_AF__Bc0055E24f | 22 | protein | chr5 |
| Ctg3364_AF__Bc0006O16r | 7 | ---NA--- | chr5 |
| Ctg352_AF__Bb0068H13r | 136 | atp citrate lyase | chr5 |
| Ctg3657_AF__Bb0053D04f | 30 | phosphatase subunit g4-1 | chr5 |
| Ctg3794_AF__Bb0038L15r | 24 | extra sporogenous cells | chr5 |
| Ctg3828_AF__Bc0061E24f | 16 | pentatricopeptide repeat-containing protein | chr5 |
| Ctg3938_AF__Bb0074A16f | 24 | dehydration-responsive expressed | chr5 |
| Ctg3969_AF__Bb0001A16r | 8 | carboxyphosphonoenolpyruvate mutase | chr5 |
| Ctg4044_AF__Bb0062H03r | 47 | lipid binding | chr5 |
| Ctg4105_AF__Bc0033K06r | 50 | vacuolar protein sorting 13c | chr5 |
| Ctg4105_AF__Bc0036B16f | 50 | vacuolar protein sorting-associated | chr5 |
| Ctg4119_AF__Bc0035L14f | 35 | methionyl-trna synthetase | chr5 |
| Ctg414_AF__Bb0014N04f | 47 | methionyl-trna synthetase | chr5 |
| Ctg456_AF__Bc0017L07r | 7 | protein | chr5 |
| Ctg4706_AF__Bb0019J09f | 42 | beta-ketoacyl- synthase | chr5 |
| Ctg493_AF__Bb0013E20f | 17 | aldehyde dehydrogenase | chr5 |
| Ctg499_AF__Bb0030D07r | 17 | protein | chr5 |
| Ctg516_AF__Bc0028I04f | 17 | mitogen-activated protein kinase | chr5 |
| Ctg518_AF__Bb0010J02r | 17 | unnamed protein product [Vitis vinifera] | chr5 |
| Ctg524_AF__Bc0072N05r | 17 | protein | chr5 |
| Ctg54_AF__Bc0024B06f | 17 | ---NA--- | chr5 |
| Ctg619_AF__Bc0063A05r | 17 | protein | chr5 |
| Ctg619_AF__Bc0069D18r | 17 | glycoside hydrolase family 28 protein polygalacturonase family protein | chr5 |
| Ctg638_AF__Bc0043D05r | 17 | thioredoxin-like protein 4a | chr5 |
| Ctg66_AF__Bc0017L17f | 17 | protein | chr5 |
| Ctg700_AF__Bb0071K18r | 17 | peptidyl-prolyl cis-trans isomerase cyclophilin-type family protein | chr5 |
| Ctg739_AF__Bc0020I22f | 17 | mitogen-activated protein kinase | chr5 |
| Ctg792_AF__Bb0011P17r | 17 | adp-glucose pyrophosphorylase large subunit | chr5 |
| Ctg802_AF__Bc0004J19r | 17 | boron transporter | chr5 |
| Ctg859_AF__Bb0033A21r | 17 | protein | chr5 |
| Ctg859_AF__Bc0050L07r | 17 | protein | chr5 |
| Ctg860_AF__Bc0065F16f | 17 | pentatricopeptide repeat-containing protein | chr5 |
| Ctg931_AF__Bb0008D03f | 17 | multidrug pheromone mdr abc transporter family | chr5 |
| Ctg931_AF__Bc0021M05f | 17 | splicing factor | chr5 |
| Ctg931_AF__Bc0041E07r | 17 | u5 snrnp-specific factor | chr5 |
| Ctg934_AF__Bc0015G04f | 17 | dna binding | chr5 |
| Ctg935_AF__Bc0032M20r | 17 | autoinhibited calcium atpase | chr5 |
| Ctg947_AF__Bc0024C01f | 17 | with no lysine kinase | chr5 |
| Ctg947_AF__Bc0058G11r | 17 | hsp70 chaperone | chr5 |
| Ctg1015_AF__Bc0014E02f | 13 | heavy metal p-type atpase | chr6 |
| Ctg1035_AF__Bc0060K17f | 16 | elongation factor 1-alpha | chr6 |
| Ctg1069_AF__Bc0052H22r | 7 | protein | chr6 |
| Ctg110_AF__Bc0018E09f | 14 | ---NA--- | chr6 |
| Ctg1108_AF__Bc0022O19r | 39 | ---NA--- | chr6 |
| Ctg1138_AF__Bb0053H18f | 11 | transferring glycosyl | chr6 |
| Ctg1151_AF__Bb0040I18f | 25 | pheophorbide a | chr6 |
| Ctg1151_AF__Bc0036B21r | 25 | protein | chr6 |
| Ctg1190_AF__Bb0066N14r | 13 | protein | chr6 |
| Ctg1190_AF__Bb0074N17f | 13 | protein | chr6 |
| Ctg1190_AF__Bc0073K05f | 13 | phosphatidylcholine: diacylglycerol acyltransferase | chr6 |
| Ctg122_AF__Bc0024M21f | 17 | rubisco subunit binding-protein alpha subunit | chr6 |
| Ctg1236_AF__Bb0045O17f | 15 | ---NA--- | chr6 |
| Ctg1239_AF__Bb0052D14f | 12 | oxysterol-binding protein | chr6 |
| Ctg1288_AF__Bc0026L23f | 23 | adenosine 3-phospho 5-phosphosulfate transporter 1 | chr6 |
| Ctg1298_AF__Bc0038J04r | 21 | ran gtpase binding | chr6 |
| Ctg1298_AF__Bc0045P03r | 21 | non-imprinted in prader-willi angelman syndrome region | chr6 |
| Ctg1598_AF__Bb0067F13f | 10 | protein | chr6 |
| Ctg167_AF__Bc0020K24f | 9 | protein | chr6 |
| Ctg1689_AF__Bb0013E01f | 7 | pleckstrin homology domain-containing protein domain-containing protein | chr6 |
| Ctg1724_AF__Bb0038A14r | 7 | atp-binding cassette | chr6 |
| Ctg176_AF__Bc0037G10r | 8 | protein | chr6 |
| Ctg1764_AF__Bc0065I06f | 7 | protein | chr6 |
| Ctg1821_AF__Bc0030K18f | 4 | protein | chr6 |
| Ctg1821_AF__Bc0044C23r | 4 | ac1147-like protein | chr6 |
| Ctg188_AF__Bc0066P22f | 5 | ac1147-like protein | chr6 |
| Ctg1894_AF__Bc0064K05f | 8 | dna binding | chr6 |
| Ctg193_AF__Bc0002N18f | 6 | PREDICTED: hypothetical protein [Vitis vinifera] | chr6 |
| Ctg2_AF__Bc0001C18r | 10 | protein | chr6 |
| Ctg2_AF__Bc0014L22r | 10 | lps-binding protein | chr6 |
| Ctg2_AF__Bc0017I16f | 10 | protein | chr6 |
| Ctg2_AF__Bc0028D12f | 10 | ac1147-like protein | chr6 |
| Ctg2_AF__Bc0037L01f | 10 | ac1147-like protein | chr6 |
| Ctg2_AF__Bc0044A01f | 10 | ac1147-like protein | chr6 |
| Ctg2_AF__Bc0052B12f | 10 | ac1147-like protein | chr6 |
| Ctg2_AF__Bc0052B12r | 10 | ac1147-like protein | chr6 |
| Ctg2_AF__Bc0052O08f | 10 | protein | chr6 |
| Ctg2_AF__Bc0053O05f | 10 | lps-binding protein | chr6 |
| Ctg2_AF__Bc0054M08r | 10 | protein | chr6 |
| Ctg2_AF__Bc0071J22r | 10 | ac1147-like protein | chr6 |
| Ctg2_AF__Bc0072I23f | 10 | lps-binding protein | chr6 |
| Ctg2_AF__Bc0073G09f | 10 | ac1147-like protein | chr6 |
| Ctg2_AF__Bc0073M04f | 10 | ac1147-like protein | chr6 |
| Ctg2_AF__Bc0075O18f | 10 | ac1147-like protein | chr6 |
| Ctg211_AF__Bb0035F24r | 4 | at3g46970 f13i12_20 | chr6 |
| Ctg216_AF__Bc0017A22r | 6 | ac1147-like protein | chr6 |
| Ctg2183_AF__Bc0003E05f | 7 | oxysterol-binding protein | chr6 |
| Ctg236_AF__Bc0012H11f | 2 | nadh dehydrogenase subunit 5 | chr6 |
| Ctg2372_AF__Bc0066J23r | 11 | gtp cyclohydrolase ii -dihydroxy-2-butanone-4-phoshate synthase | chr6 |
| Ctg2445_AF__Bc0006D19r | 5 | flavin-containing monooxygenase family protein fmo family protein | chr6 |
| Ctg298_AF__Bb0043N19f | 2 | cax1 (cation exchanger 1) calcium ion transmembrane transporter calcium:cation antiporter calcium:hydrogen antiporter | chr6 |
| Ctg2992_AF__Bc0037N20r | 9 | protein | chr6 |
| Ctg2992_AF__Bc0038A18f | 9 | lps-binding protein | chr6 |
| Ctg310_AF__Bc0028L11f | 4 | protein | chr6 |
| Ctg315_AF__Bb0005H18r | 2 | 10-formyltetrahydrofolate synthetase | chr6 |
| Ctg321_AF__Bb0028M15r | 5 | retrotransposon ty1-copia subclass | chr6 |
| Ctg3265_AF__Bc0012M10r | 128 | lps-binding protein | chr6 |
| Ctg3265_AF__Bc0048N20f | 128 | ac1147-like protein | chr6 |
| Ctg3442_AF__Bb0077M24r | 494 | atp synthase cf0 subunit i protein | chr6 |
| Ctg346_AF__Bc0035B02f | 20 | ac1147-like protein | chr6 |
| Ctg3482_AF__Bc0033D22r | 6 | cucm1_cucme ame: full=cucumisin ame: allergen=cuc m 1 flags: precursor | chr6 |
| Ctg3511_AF__Bb0015E12r | 62 | atp-binding cassette | chr6 |
| Ctg355_AF__Bb0013K08f | 12 | protein | chr6 |
| Ctg3591_AF__Bb0009K04f | 64 | protein | chr6 |
| Ctg3622_AF__Bb0048G10r | 31 | hypothetical protein SORBIDRAFT_01g011010 [Sorghum bicolor] | chr6 |
| Ctg369_AF__Bc0010D17r | 2 | ---NA--- | chr6 |
| Ctg378_AF__Bc0041H02r | 22 | endo excinuclease amino terminal domain-containing protein | chr6 |
| Ctg38_AF__Bc0060A21r | 30 | lps-binding protein | chr6 |
| Ctg3835_AF__Bc0057J19f | 13 | polyketide synthase | chr6 |
| Ctg3836_AF__Bc0047D05r | 22 | unnamed protein product [Vitis vinifera] | chr6 |
| Ctg392_AF__Bb0004F23f | 14 | nuclear matrix protein 1 | chr6 |
| Ctg404_AF__Bb0041G20f | 2 | protein | chr6 |
| Ctg404_AF__Bb0050N08f | 2 | heat repeat family expressed | chr6 |
| Ctg4409_AF__Bc0067K07f | 43 | ---NA--- | chr6 |
| Ctg4631_AF__Bb0019L01f | 27 | serine threonine protein | chr6 |
| Ctg4631_AF__Bc0022C14r | 27 | alkaline neutral invertase | chr6 |
| Ctg4649_AF__Bb0010I02f | 23 | xanthine dehydrogenase | chr6 |
| Ctg470_AF__Bc0042J11r | 2 | dna-damage-inducible protein | chr6 |
| Ctg4733_AF__Bb0043L21f | 139 | endoplasmic reticulum | chr6 |
| Ctg489_AF__Bc0049D01f | 17 | PREDICTED: hypothetical protein [Vitis vinifera] | chr6 |
| Ctg524_AF__Bc0055K21r | 17 | protein | chr6 |
| Ctg524_AF__Bc0072N05f | 17 | ac1147-like protein | chr6 |
| Ctg561_AF__Bb0071C14f | 17 | protein | chr6 |
| Ctg583_AF__Bb0039E18f | 17 | ---NA--- | chr6 |
| Ctg583_AF__Bb0072C12r | 17 | 2-phosphoglycerate kinase-related | chr6 |
| Ctg598_AF__Bb0066E13f | 17 | pentatricopeptide repeat-containing | chr6 |
| Ctg617_AF__Bc0007E09f | 17 | ubiquitin-activating enzyme e1 expressed | chr6 |
| Ctg674_AF__Bc0019K01f | 17 | ---NA--- | chr6 |
| Ctg675_AF__Bc0070K09f | 17 | ---NA--- | chr6 |
| Ctg753_AF__Bb0027G15f | 17 | ---NA--- | chr6 |
| Ctg768_AF__Bc0008A13r | 17 | ac1147-like protein | chr6 |
| Ctg776_AF__Bb0006H03r | 17 | ---NA--- | chr6 |
| Ctg838_AF__Bc0043B15r | 17 | predicted protein [Populus trichocarpa] | chr6 |
| Ctg843_AF__Bb0061C01r | 17 | protein | chr6 |
| Ctg843_AF__Bb0065O09r | 17 | protein | chr6 |
| Ctg843_AF__Bc0030H21f | 17 | glycosyl transferase family 2 | chr6 |
| Ctg890_AF__Bc0021I24f | 17 | chitinase 1 | chr6 |
| Ctg890_AF__Bc0021I24r | 17 | ---NA--- | chr6 |
| Ctg957_AF__Bc0049B18r | 17 | ---NA--- | chr6 |
| Ctg972_AF__Bb0064N19r | 17 | sorting and assembly machinery | chr6 |
| Ctg1148_AF__Bc0049M07f | 8 | glucan endo- -beta-glucosidase | chr7 |
| Ctg1195_AF__Bc0027F03r | 20 | AC007138_14predicted protein of unknown function [Arabidopsis thaliana] | chr7 |
| Ctg1222_AF__Bb0046B08f | 34 | predicted protein [Populus trichocarpa] | chr7 |
| Ctg1237_AF__Bb0029P08f | 40 | protein | chr7 |
| Ctg1269_AF__Bb0046F18f | 51 | unnamed protein product [Vitis vinifera] | chr7 |
| Ctg1362_AF__Bb0014N10r | 9 | helicase domain-containing protein | chr7 |
| Ctg1434_AF__Bc0071G04f | 27 | endonuclease exonuclease phosphatase family protein | chr7 |
| Ctg1477_AF__Bc0017L16r | 18 | atp binding | chr7 |
| Ctg1596_AF__Bb0074B14f | 37 | protein | chr7 |
| Ctg1622_AF__Bb0055I16r | 11 | ap2 erf domain-containing transcription factor | chr7 |
| Ctg1630_AF__Bb0017H20r | 27 | protein | chr7 |
| Ctg1630_AF__Bb0052I10f | 27 | protein | chr7 |
| Ctg1642_AF__Bc0046F09r | 7 | protein | chr7 |
| Ctg1728_AF__Bc0043F08r | 10 | zinc ion binding | chr7 |
| Ctg1825_AF__Bc0059K22r | 6 | phosphate transporter | chr7 |
| Ctg2015_AF__Bb0054M07f | 5 | protein | chr7 |
| Ctg2502_AF__Bb0036D04r | 5 | rna binding | chr7 |
| Ctg275_AF__Bc0048M15r | 2 | bglu11 (beta glucosidase 11) catalytic cation binding hydrolyzing o-glycosyl compounds | chr7 |
| Ctg278_AF__Bb0050A14f | 3 | ---NA--- | chr7 |
| Ctg2825_AF__Bc0051F06f | 6 | cbs domain-containing | chr7 |
| Ctg309_AF__Bc0047D16r | 2 | homocysteine s-methyltransferase 3 | chr7 |
| Ctg320_AF__Bb0027N20f | 5 | beta- | chr7 |
| Ctg320_AF__Bb0027N20r | 5 | ---NA--- | chr7 |
| Ctg320_AF__Bc0021H02r | 5 | sucrose synthase | chr7 |
| Ctg340_AF__Bb0010J15r | 2 | stachyose synthase | chr7 |
| Ctg3591_AF__Bb0004L03f | 64 | gag-protease-integrase-rt-r polyprotein | chr7 |
| Ctg3591_AF__Bb0067F03f | 64 | condensin complex component | chr7 |
| Ctg365_AF__Bb0015C13f | 2 | protein | chr7 |
| Ctg365_AF__Bb0032P09r | 2 | predicted protein [Populus trichocarpa] | chr7 |
| Ctg3678_AF__Bc0039H06r | 10 | rootless concerning crown and seminal lateral roots | chr7 |
| Ctg3769_AF__Bb0005J24f | 9 | protein | chr7 |
| Ctg39_AF__Bc0025H08f | 3 | gag-protease-integrase-rt-r polyprotein | chr7 |
| Ctg393_AF__Bc0055O15f | 27 | protein | chr7 |
| Ctg4152_AF__Bc0012A18r | 27 | multidrug resistance protein abc transporter family | chr7 |
| Ctg512_AF__Bb0050B18f | 17 | protein | chr7 |
| Ctg54_AF__Bb0054J11f | 17 | alcohol dehydrogenase class iii | chr7 |
| Ctg54_AF__Bb0077M21f | 17 | glutathione-dependent formaldehyde dehydrogenase | chr7 |
| Ctg629_AF__Bb0037E07f | 17 | ---NA--- | chr7 |
| Ctg629_AF__Bc0067I18r | 17 | tetratricopeptide repeat | chr7 |
| Ctg639_AF__Bc0039F02f | 17 | myosin xi | chr7 |
| Ctg65_AF__Bb0019C08f | 17 | protein | chr7 |
| Ctg72_AF__Bc0044K23r | 17 | predicted protein [Physcomitrella patens subsp. patens] | chr7 |
| Ctg771_AF__Bb0033M01r | 17 | pectinesterase family protein | chr7 |
| Ctg771_AF__Bc0023P06f | 17 | protein | chr7 |
| Ctg808_AF__Bb0057F09r | 17 | rsh1 (rela-spot homolog 1) catalytic | chr7 |
| Ctg892_AF__Bc0044E23r | 17 | protein | chr7 |
| Ctg94_AF__Bc0041J19r | 17 | catalytic hydrolase | chr7 |
| Ctg194_AF__Bb0056J15f | 19 | heat shock protein 70 -interacting | chr7_random |
| Ctg1004_AF__Bb0015M06r | 24 | calcium-dependent protein | chr8 |
| Ctg1067_AF__Bb0062M18r | 32 | cytochrome p450 | chr8 |
| Ctg1067_AF__Bc0021D21f | 32 | pectinesterase family protein | chr8 |
| Ctg1067_AF__Bc0040M11r | 32 | cytochrome p450 | chr8 |
| Ctg1090_AF__Bb0006H22r | 23 | polyribonucleotide nucleotidyltransferase (polynucleotide phosphorylase) | chr8 |
| Ctg1151_AF__Bc0038L07f | 25 | protein | chr8 |
| Ctg1219_AF__Bb0020G18f | 17 | rna polymerase ii transcription factor protein binding transcription activator transcription regulator translation initiation factor zinc ion binding | chr8 |
| Ctg122_AF__Bc0009M23f | 17 | protein | chr8 |
| Ctg1343_AF__Bb0030L19r | 15 | iil1 (isopropyl malate isomerase large subunit 1) 4 4 sulfur cluster binding hydro-lyase lyase | chr8 |
| Ctg142_AF__Bc0072I01f | 5 | protein | chr8 |
| Ctg1546_AF__Bb0030E12r | 20 | exostosin-like glycosyltransferase | chr8 |
| Ctg1546_AF__Bb0035H06r | 20 | dna helicase | chr8 |
| Ctg1546_AF__Bc0024B11r | 20 | abc transporter cholesterol phospholipid flippase | chr8 |
| Ctg1580_AF__Bc0044L23r | 5 | protein | chr8 |
| Ctg1626_AF__Bb0029M21r | 9 | ORF47a [Pinus koraiensis] | chr8 |
| Ctg1677_AF__Bc0070F01r | 6 | uncoupling protein | chr8 |
| Ctg1792_AF__Bb0066G05f | 12 | atp binding protein | chr8 |
| Ctg1907_AF__Bb0037N04r | 10 | nucleotide binding | chr8 |
| Ctg191_AF__Bc0039J24r | 6 | protein | chr8 |
| Ctg2092_AF__Bc0043H08r | 6 | ---NA--- | chr8 |
| Ctg213_AF__Bc0045F22r | 4 | gamma-glutamyl phosphate reductase | chr8 |
| Ctg224_AF__Bc0040K13f | 5 | beta-d-glucan exohydrolase | chr8 |
| Ctg2303_AF__Bb0046K02f | 6 | amp deaminase | chr8 |
| Ctg246_AF__Bb0053P20f | 5 | slt1 protein | chr8 |
| Ctg2557_AF__Bc0003G07f | 2 | pentatricopeptide repeat-containing | chr8 |
| Ctg284_AF__Bb0051L19f | 11 | heat shock protein 70 | chr8 |
| Ctg291_AF__Bb0024L14f | 2 | plastid division protein | chr8 |
| Ctg291_AF__Bb0040P06f | 2 | f-box family protein | chr8 |
| Ctg298_AF__Bc0040L19f | 2 | chlorophyll a b-binding protein cp29 | chr8 |
| Ctg307_AF__Bb0006D05r | 2 | starch branching enzyme 3 | chr8 |
| Ctg3552_AF__Bc0054H18f | 51 | protein | chr8 |
| Ctg3622_AF__Bc0018D18f | 31 | protein | chr8 |
| Ctg3695_AF__Bc0051M17r | 45 | rna polymerase i largest subunit | chr8 |
| Ctg378_AF__Bb0074C23f | 22 | myosin heavy chain-like | chr8 |
| Ctg3836_AF__Bb0014C08r | 22 | c2 domain-containing protein | chr8 |
| Ctg3836_AF__Bc0002B15f | 22 | emb2735 (embryo defective 2735) | chr8 |
| Ctg3859_AF__Bb0024N20r | 35 | dna binding protein | chr8 |
| Ctg3859_AF__Bc0041N13f | 35 | protein | chr8 |
| Ctg3990_AF__Bb0055G15f | 2 | pyruvate kinase family expressed | chr8 |
| Ctg404_AF__Bc0067B21f | 2 | calcium calmodulin-dependent protein kinase | chr8 |
| Ctg4067_AF__Bb0040G07f | 23 | hypothetical protein [Vitis vinifera] | chr8 |
| Ctg419_AF__Bb0070I19r | 2 | hsc70-1 (heat shock cognate 70 kda protein 1) atp binding isoform 2 | chr8 |
| Ctg4349_AF__Bc0026G15f | 13 | nodulation receptor kinase | chr8 |
| Ctg447_AF__Bb0006A16r | 2 | polynucleotidyl ribonuclease h fold | chr8 |
| Ctg452_AF__Bc0016K14f | 9 | _rbz domain protein | chr8 |
| Ctg4649_AF__Bb0053M18f | 23 | receptor-like protein kinase | chr8 |
| Ctg489_AF__Bc0038P19f | 17 | atp binding | chr8 |
| Ctg497_AF__Bc0025O12f | 17 | ---NA--- | chr8 |
| Ctg545_AF__Bc0073B20r | 17 | ---NA--- | chr8 |
| Ctg567_AF__Bb0072P11r | 17 | secondary cell wall-related glycosyltransferase family 47 | chr8 |
| Ctg567_AF__Bc0035F13f | 17 | protein | chr8 |
| Ctg596_AF__Bb0005I11r | 17 | pyrroline-5-carboxylate synthetase | chr8 |
| Ctg60_AF__Bb0051P15f | 17 | ---NA--- | chr8 |
| Ctg604_AF__Bc0050M22r | 17 | cell division protein | chr8 |
| Ctg617_AF__Bb0028I10f | 17 | ---NA--- | chr8 |
| Ctg617_AF__Bb0028I10r | 17 | ubiquitin-activating enzyme | chr8 |
| Ctg629_AF__Bc0066B01r | 17 | pentatricopeptide repeat-containing | chr8 |
| Ctg648_AF__Bc0042H03f | 17 | protein | chr8 |
| Ctg650_AF__Bc0006N10r | 17 | adenylosuccinate synthetase | chr8 |
| Ctg753_AF__Bb0010H04f | 17 | predicted protein [Populus trichocarpa] | chr8 |
| Ctg859_AF__Bb0019I10r | 17 | eukaryotic initiation factor 3e | chr8 |
| Ctg861_AF__Bb0041K17f | 17 | polynucleotide phosphorylase polyadenylase | chr8 |
| Ctg861_AF__Bc0032O04f | 17 | glycosyl hydrolase family 17 protein | chr8 |
| Ctg861_AF__Bc0032O04r | 17 | cationic amino acid transporter | chr8 |
| Ctg888_AF__Bb0048G19r | 17 | mitochondrial respiratory chain complexes assembly protein | chr8 |
| Ctg900_AF__Bc0059C11r | 17 | uxs1 (udp-glucuronic acid decarboxylase 1) udp-glucuronate decarboxylase catalytic | chr8 |
| Ctg94_AF__Bb0058F14r | 17 | pre-rrna processing protein rrp5 | chr8 |
| Ctg95_AF__Bb0029E09f | 17 | ---NA--- | chr8 |
| Ctg98_AF__Bb0034H14f | 17 | adenylyl-sulfate kinase | chr8 |
| Ctg1069_AF__Bc0019D24f | 7 | mms19 nucleotide excision repair homolog | chr9 |
| Ctg1078_AF__Bc0052K14r | 20 | rna m5u | chr9 |
| Ctg1161_AF__Bc0064C18f | 11 | protein | chr9 |
| Ctg1234_AF__Bb0029J24f | 59 | autoinhibited calcium atpase | chr9 |
| Ctg1284_AF__Bb0063P22r | 27 | uv excision repair protein | chr9 |
| Ctg1700_AF__Bb0007B03r | 17 | plastid ppgpp synthase | chr9 |
| Ctg1700_AF__Bc0027P22f | 17 | type 2a protein serine threonine phosphatase 55 kda b regulatory subunit | chr9 |
| Ctg1717_AF__Bc0066P04f | 11 | peptide deformylase | chr9 |
| Ctg191_AF__Bb0073E14f | 6 | atp synthase alpha subunit | chr9 |
| Ctg192_AF__Bc0068O19f | 3 | pentatricopeptide repeat-containing | chr9 |
| Ctg2541_AF__Bc0030M07r | 57 | mrna splicing protein prp8 | chr9 |
| Ctg278_AF__Bb0073G07f | 3 | mannosyltransferase-like protein | chr9 |
| Ctg278_AF__Bc0042O09r | 3 | mannosyltransferase-like protein | chr9 |
| Ctg328_AF__Bc0019M12r | 2 | protein | chr9 |
| Ctg365_AF__Bc0069B02r | 2 | ribosomal protein s14p s29e containing expressed | chr9 |
| Ctg3701_AF__Bb0001O19f | 13 | mannosyltransferase-like protein | chr9 |
| Ctg441_AF__Bc0021A04r | 43 | atp-binding cassette | chr9 |
| Ctg471_AF__Bb0027N17r | 42 | ---NA--- | chr9 |
| Ctg488_AF__Bc0044F24f | 17 | retrotransposon ty1-copia subclass | chr9 |
| Ctg49_AF__Bc0040D21f | 17 | rna polymerase beta subunit | chr9 |
| Ctg517_AF__Bb0009G21r | 17 | auxin-independent growth promoter | chr9 |
| Ctg528_AF__Bb0072J23f | 17 | ---NA--- | chr9 |
| Ctg595_AF__Bb0059A14r | 17 | at3g14120 mag2_7 | chr9 |
| Ctg715_AF__Bc0073O21r | 17 | ring-h2 zinc finger protein | chr9 |
| Ctg767_AF__Bc0062P15f | 17 | regulator of telomere elongation helicase 1 | chr9 |
| Ctg870_AF__Bc0075B03f | 17 | pdr-like abc transporter | chr9 |
| Ctg89_AF__Bb0064A16f | 17 | adenine nucleotide translocase | chr9 |
| Ctg987_AF__Bc0011H24f | 17 | ---NA--- | chr9 |
| Ctg1062_AF__Bb0015E01f | 10 | vacuolar protein | chr10 |
| Ctg1114_AF__Bc0041H22f | 9 | magnesium chelatase subunit i | chr10 |
| Ctg1563_AF__Bc0046H10r | 79 | erd4 protein | chr10 |
| Ctg16_AF__Bb0010B16f | 131 | protein | chr10 |
| Ctg193_AF__Bb0037K07r | 6 | integral membrane protein | chr10 |
| Ctg1984_AF__Bc0008D10r | 7 | uv-damaged dna-binding | chr10 |
| Ctg2222_AF__Bc0011O16f | 21 | protein | chr10 |
| Ctg255_AF__Bb0011N09f | 2 | protein | chr10 |
| Ctg2570_AF__Bc0072F22f | 4 | trypsin domain-containing | chr10 |
| Ctg3442_AF__Bb0035M18r | 494 | ribosomal protein s2 | chr10 |
| Ctg3690_AF__Bb0025H20f | 29 | 3-hydroxyisobutyrate dehydrogenase | chr10 |
| Ctg4533_AF__Bc0033L06f | 29 | wrky transcription | chr10 |
| Ctg1018_AF__Bb0001E15f | 23 | protein | chr10_random |
| Ctg202_AF__Bb0018B12r | 4 | ---NA--- | chr10_random |
| Ctg212_AF__Bc0065G09r | 5 | ---NA--- | chr10_random |
| Ctg229_AF__Bb0021M04f | 3 | ---NA--- | chr10_random |
| Ctg263_AF__Bc0048L24f | 2 | receptor-like kinase | chr10_random |
| Ctg3574_AF__Bb0036M07r | 24 | ---NA--- | chr10_random |
| Ctg379_AF__Bb0018J16r | 4 | ---NA--- | chr10_random |
| Ctg3828_AF__Bb0038L04r | 16 | ---NA--- | chr10_random |
| Ctg437_AF__Bb0024F11f | 26 | glutaredoxin s17 | chr10_random |
| Ctg639_AF__Bb0010H19r | 17 | mate efflux family expressed | chr10_random |
| Ctg639_AF__Bc0017K02r | 17 | protein | chr10_random |
| Ctg1004_AF__Bb0047O10r | 24 | glycosyl transferase family 17 protein | chr11 |
| Ctg113_AF__Bc0026L16f | 124 | auxin response factor 7b | chr11 |
| Ctg113_AF__Bc0043P15f | 124 | protein kinase | chr11 |
| Ctg1152_AF__Bc0051H19r | 15 | protein | chr11 |
| Ctg1234_AF__Bb0073M17r | 31 | autoinhibited calcium atpase | chr11 |
| Ctg1234_AF__Bc0059J08f | 31 | ---NA--- | chr11 |
| Ctg1256_AF__Bc0042J22r | 38 | nuclear protein | chr11 |
| Ctg1278_AF__Bb0015N21f | 30 | protein | chr11 |
| Ctg1284_AF__Bc0041L14r | 11 | hhp2 (heptahelical transmembrane protein2) receptor | chr11 |
| Ctg1574_AF__Bb0077J15f | 79 | ---NA--- | chr11 |
| Ctg185_AF__Bb0047C15f | 20 | protein | chr11 |
| Ctg2_AF__Bc0001C18f | 10 | protein | chr11 |
| Ctg2_AF__Bc0011G18r | 10 | gamma 1 | chr11 |
| Ctg2_AF__Bc0014L22f | 10 | gamma 1 | chr11 |
| Ctg2_AF__Bc0064N06f | 10 | protein | chr11 |
| Ctg218_AF__Bb0017A06f | 6 | nucleotide binding | chr11 |
| Ctg2537_AF__Bc0074M02f | 15 | protein | chr11 |
| Ctg2601_AF__Bb0059H02f | 5 | hcf101 (high-chlorophyll-fluorescence 101) atp binding | chr11 |
| Ctg2634_AF__Bc0015N10r | 6 | protein arginine n- | chr11 |
| Ctg285_AF__Bb0028D22r | 2 | protein | chr11 |
| Ctg31_AF__Bb0017G16r | 4 | nac domain ipr003441 | chr11 |
| Ctg3442_AF__Bc0010J05r | 494 | acrs-like protein | chr11 |
| Ctg3486_AF__Bb0019N12f | 79 | protein | chr11 |
| Ctg3591_AF__Bc0075A15f | 64 | atp-binding cassette | chr11 |
| Ctg36_AF__Bb0053A11r | 17 | gag-protease-integrase-rt-r polyprotein | chr11 |
| Ctg38_AF__Bc0060A21f | 30 | protein | chr11 |
| Ctg386_AF__Bb0067E22r | 35 | syringolide-induced protein 19-1-5 | chr11 |
| Ctg404_AF__Bb0059P12f | 2 | digalactosyldiacylglycerol synthase expressed | chr11 |
| Ctg43_AF__Bb0043N02r | 6 | protein | chr11 |
| Ctg441_AF__Bb0047M02r | 43 | protein | chr11 |
| Ctg483_AF__Bc0043E14r | 17 | ztl | chr11 |
| Ctg522_AF__Bb0023L14r | 17 | aspartate aminotransferase | chr11 |
| Ctg524_AF__Bc0065L12f | 17 | protein | chr11 |
| Ctg550_AF__Bb0014I16r | 17 | protein | chr11 |
| Ctg568_AF__Bc0010J01f | 17 | cycloartenol synthase | chr11 |
| Ctg639_AF__Bb0041G09f | 17 | protein | chr11 |
| Ctg678_AF__Bc0063H09f | 17 | the saccharomyces cerevisiae sec7 ank accession number j03918 | chr11 |
| Ctg715_AF__Bc0073O21f | 17 | sec34-like family protein | chr11 |
| Ctg787_AF__Bc0004F19f | 17 | kinesin-like protein | chr11 |
| Ctg898_AF__Bb0064F24f | 17 | glycoside hydrolase family 28 protein polygalacturonase family protein | chr11 |
| Ctg92_AF__Bb0011P08r | 17 | gag-protease-integrase-rt-r polyprotein | chr11 |
| Ctg994_AF__Bb0022A08f | 17 | ankyrin repeat-containing | chr11 |
| Ctg288_AF__Bc0011A04r | 3 | zn cd p -type atpase | chr11_random |
| Ctg424_AF__Bb0046E10f | 16 | sucrose phosphate synthase | chr11_random |
| Ctg424_AF__Bb0059H15f | 16 | sucrose phosphate synthase | chr11_random |
| Ctg1001_AF__Bb0013I11r | 48 | protein | chr12 |
| Ctg1019_AF__Bb0075G02r | 44 | phosphoenol pyruvate carboxykinase | chr12 |
| Ctg1101_AF__Bc0014N09r | 24 | serine-threonine protein plant- | chr12 |
| Ctg13_AF__Bc0019L13f | 27 | protein | chr12 |
| Ctg1321_AF__Bc0041F05f | 8 | nucleobase ascorbate transporter | chr12 |
| Ctg1460_AF__Bb0020I08r | 13 | gh3 family protein | chr12 |
| Ctg1605_AF__Bb0074H08r | 93 | chlorophyll a b binding protein | chr12 |
| Ctg1722_AF__Bc0017H16f | 37 | cmv 1a interacting protein 1 | chr12 |
| Ctg1835_AF__Bc0030O07r | 36 | transferring glycosyl | chr12 |
| Ctg226_AF__Bc0068P09r | 26 | transferring glycosyl | chr12 |
| Ctg243_AF__Bb0026B07f | 3 | vacuolar protein sorting | chr12 |
| Ctg243_AF__Bc0024D09f | 3 | f-box family protein | chr12 |
| Ctg3580_AF__Bb0045E03r | 14 | rhomboid family protein | chr12 |
| Ctg3645_AF__Bb0017N18r | 40 | at1g06520-like protein | chr12 |
| Ctg3690_AF__Bc0065O09r | 29 | protein | chr12 |
| Ctg385_AF__Bc0004F24f | 6 | ---NA--- | chr12 |
| Ctg39_AF__Bb0023I06f | 3 | ebs-bah-phd domain-containing protein | chr12 |
| Ctg39_AF__Bb0065O01f | 3 | hexose transporter | chr12 |
| Ctg3948_AF__Bb0060J03f | 22 | rhomboid family protein | chr12 |
| Ctg431_AF__Bc0043J03f | 4 | (1-4)-beta-mannan endohydrolase | chr12 |
| Ctg4324_AF__Bc0029M04f | 11 | 3-isopropylmalate dehydrogenase | chr12 |
| Ctg462_AF__Bc0061H19f | 29 | protein | chr12 |
| Ctg477_AF__Bc0043J15f | 17 | protein | chr12 |
| Ctg538_AF__Bb0018G12r | 17 | pentatricopeptide repeat-containing | chr12 |
| Ctg541_AF__Bc0028A01f | 17 | protein | chr12 |
| Ctg584_AF__Bc0050H01f | 17 | protein | chr12 |
| Ctg68_AF__Bc0035N22r | 17 | atp phosphoribosyl transferase | chr12 |
| Ctg693_AF__Bc0024M24f | 17 | heterogeneous nuclear ribonucleoprotein | chr12 |
| Ctg713_AF__Bb0027F11r | 17 | formin homology 2 domain-containing protein 5 | chr12 |
| Ctg826_AF__Bb0056P04r | 17 | prli-interacting factor k | chr12 |
| Ctg854_AF__Bb0060G22r | 17 | legumin-like protein | chr12 |
| Ctg949_AF__Bb0013M20f | 17 | receptor protein | chr12 |
| Ctg99_AF__Bb0021E15f | 17 | pentatricopeptide repeat-containing protein | chr12 |
| Ctg997_AF__Bb0033C12r | 17 | vacuolar membrane protein | chr12 |
| Ctg179_AF__Bc0043I20r | 7 | PREDICTED: hypothetical protein [Vitis vinifera] | chr12_random |
| Ctg253_AF__Bb0052M13f | 2 | retrotransposon ty1-copia subclass | chr12_random |
| Ctg1018_AF__Bc0002F13r | 23 | gag-protease-integrase-rt-r polyprotein | chr13 |
| Ctg1110_AF__Bb0039P02f | 20 | protein | chr13 |
| Ctg112_AF__Bc0026F05f | 15 | in2-1 protein | chr13 |
| Ctg1142_AF__Bc0037D20r | 9 | regulator of nonsense | chr13 |
| Ctg1154_AF__Bb0030N23r | 9 | acyl- binding | chr13 |
| Ctg1184_AF__Bc0044M21r | 21 | protein | chr13 |
| Ctg1192_AF__Bb0048L07f | 9 | aux1-like permease | chr13 |
| Ctg1441_AF__Bb0001L17f | 14 | egy2 metalloendopeptidase | chr13 |
| Ctg1721_AF__Bc0055O13r | 12 | conserved hypothetical protein [Ricinus communis] | chr13 |
| Ctg1864_AF__Bc0005H19f | 10 | notchless-like protein | chr13 |
| Ctg192_AF__Bc0068O19r | 3 | clathrin heavy | chr13 |
| Ctg2005_AF__Bb0038P17f | 6 | 2-oxoglutarate e1 component | chr13 |
| Ctg2070_AF__Bc0057M09f | 7 | ---NA--- | chr13 |
| Ctg213_AF__Bc0045F22f | 4 | vacuolar protein | chr13 |
| Ctg2247_AF__Bc0069A19f | 6 | pyruvate dehydrogenase component x | chr13 |
| Ctg246_AF__Bc0049N10r | 5 | ---NA--- | chr13 |
| Ctg277_AF__Bb0024D18f | 3 | ---NA--- | chr13 |
| Ctg311_AF__Bb0018A23r | 3 | copia-like polyprotein | chr13 |
| Ctg3259_AF__Bc0030O18r | 3 | ---NA--- | chr13 |
| Ctg3503_AF__Bb0013E04r | 60 | retrotransposon unclassified | chr13 |
| Ctg356_AF__Bc0037A06f | 109 | plastid dna-binding protein | chr13 |
| Ctg3607_AF__Bb0053F18f | 93 | PREDICTED: hypothetical protein [Vitis vinifera] | chr13 |
| Ctg3679_AF__Bc0040B05f | 19 | protein | chr13 |
| Ctg3836_AF__Bc0011A06f | 22 | protein | chr13 |
| Ctg3859_AF__Bb0073F04r | 35 | ---NA--- | chr13 |
| Ctg404_AF__Bb0029D08f | 2 | nedd1 (neural precursor cell developmentally down-regulated gene 1) nucleotide binding | chr13 |
| Ctg446_AF__Bc0052I18r | 2 | gag-protease-integrase-rt-r polyprotein | chr13 |
| Ctg4649_AF__Bc0044E05f | 23 | protein | chr13 |
| Ctg4684_AF__Bb0024N10r | 64 | ---NA--- | chr13 |
| Ctg516_AF__Bb0070L24f | 17 | protein | chr13 |
| Ctg583_AF__Bb0034G13f | 17 | protein | chr13 |
| Ctg616_AF__Bb0043G18f | 17 | laccase (diphenol oxidase) | chr13 |
| Ctg616_AF__Bc0030D08r | 17 | receptor-like kinase | chr13 |
| Ctg843_AF__Bc0044P13r | 17 | protein binding | chr13 |
| Ctg97_AF__Bb0012M01r | 17 | ctr1-like protein kinase | chr13 |
| Ctg3_AF__Bb0040P24r | 7 | dead box atp-dependent rna | chr13_random |
| Ctg404_AF__Bc0035P16r | 2 | ---NA--- | chr13_random |
| Ctg1020_AF__Bb0048K03f | 11 | at5g27210 t21b4_120 | chr14 |
| Ctg1029_AF__Bc0018O08f | 22 | glutathione synthetase | chr14 |
| Ctg1051_AF__Bb0034H08f | 26 | glyceraldehyde-3-phosphate dehydrogenase | chr14 |
| Ctg1109_AF__Bc0050N21r | 28 | PREDICTED: hypothetical protein [Vitis vinifera] | chr14 |
| Ctg1167_AF__Bb0012C21r | 12 | cystathionine gamma-synthase | chr14 |
| Ctg1205_AF__Bc0061F11f | 14 | ---NA--- | chr14 |
| Ctg1205_AF__Bc0061F11r | 14 | aldehyde dehydrogenase | chr14 |
| Ctg1212_AF__Bb0063D23r | 25 | glycosyl transferase family 17 protein | chr14 |
| Ctg1222_AF__Bc0012I02r | 34 | respiratory burst | chr14 |
| Ctg156_AF__Bb0012F19r | 6 | dead box atp-dependent rna | chr14 |
| Ctg1577_AF__Bb0077E06f | 65 | 14-3-3-like protein | chr14 |
| Ctg1602_AF__Bb0017F08r | 9 | atp binding | chr14 |
| Ctg163_AF__Bc0010E21r | 5 | ---NA--- | chr14 |
| Ctg1657_AF__Bc0043F07r | 7 | mdr-like p-glycoprotein | chr14 |
| Ctg1728_AF__Bb0064G19f | 10 | general substrate transporter | chr14 |
| Ctg1738_AF__Bb0031F11r | 2 | glycosyl transferase family 17 protein | chr14 |
| Ctg187_AF__Bb0027B14r | 3 | atcfm2 (crm family member 2) rna binding | chr14 |
| Ctg187_AF__Bb0057M07r | 3 | ---NA--- | chr14 |
| Ctg2140_AF__Bb0052M18f | 42 | nica_arath ame: full=nicastrin flags: precursor | chr14 |
| Ctg2140_AF__Bc0069L06r | 42 | shaker-like potassium channel | chr14 |
| Ctg216_AF__Bb0002L19f | 6 | sialyltransferase like protein | chr14 |
| Ctg216_AF__Bc0041J11r | 6 | protein | chr14 |
| Ctg2619_AF__Bc0052E06f | 9 | atp-dependent rna helicase | chr14 |
| Ctg283_AF__Bc0060C12f | 2 | ---NA--- | chr14 |
| Ctg324_AF__Bc0050B20r | 2 | suppressor of 3-like 1 ( cerevisiae) | chr14 |
| Ctg342_AF__Bb0019I08f | 4 | lysosomal alpha- | chr14 |
| Ctg344_AF__Bb0056F02f | 3 | protein | chr14 |
| Ctg3498_AF__Bc0021I23r | 48 | serine palmitoyltransferase | chr14 |
| Ctg357_AF__Bc0038E04f | 28 | adp-ribosylation factor | chr14 |
| Ctg3765_AF__Bb0025M06f | 21 | flagellar biosynthesis protein expressed | chr14 |
| Ctg385_AF__Bc0022G21f | 6 | ---NA--- | chr14 |
| Ctg3861_AF__Bb0031I03r | 14 | mitogen-activated protein kinase | chr14 |
| Ctg3891_AF__Bb0026H09r | 23 | protein | chr14 |
| Ctg4017_AF__Bc0051L20f | 5 | unknown [Medicago truncatula] | chr14 |
| Ctg405_AF__Bc0059D22f | 2 | retrotransposon ty1-copia subclass | chr14 |
| Ctg4142_AF__Bc0062D13f | 3 | dead box atp-dependent rna | chr14 |
| Ctg4216_AF__Bb0040B23r | 11 | zinc zz-type zinc c2h2-type | chr14 |
| Ctg4227_AF__Bb0053E18r | 16 | pentatricopeptide repeat-containing | chr14 |
| Ctg44_AF__Bc0006O18f | 7 | sialyltransferase like protein | chr14 |
| Ctg45_AF__Bb0019L20f | 9 | at1g12930 f13k23_14 | chr14 |
| Ctg4706_AF__Bc0064F22r | 42 | ---NA--- | chr14 |
| Ctg471_AF__Bb0034O05r | 42 | ribose-5-phosphate isomerase | chr14 |
| Ctg4733_AF__Bb0075H14f | 139 | gag-pol identical | chr14 |
| Ctg505_AF__Bb0061I20f | 17 | ---NA--- | chr14 |
| Ctg518_AF__Bc0038J19f | 17 | metalloprotease m41 | chr14 |
| Ctg537_AF__Bb0055F20f | 17 | PREDICTED: similar to predicted protein [Acyrthosiphon pisum] | chr14 |
| Ctg537_AF__Bc0033A07r | 17 | udp-glucose dehydrogenase | chr14 |
| Ctg537_AF__Bc0053N24f | 17 | at3g29240 mxo21_9 | chr14 |
| Ctg537_AF__Bc0066L02r | 17 | unnamed protein product [Vitis vinifera] | chr14 |
| Ctg580_AF__Bb0057G10f | 17 | protein | chr14 |
| Ctg604_AF__Bb0023F12f | 17 | glutamyl-trna synthetase | chr14 |
| Ctg611_AF__Bc0009I15r | 17 | 40s ribosomal protein s3a | chr14 |
| Ctg65_AF__Bb0032K05r | 17 | vacuolar atp synthase subunit c | chr14 |
| Ctg654_AF__Bc0064H13f | 17 | at1g10410 f14n23_31 | chr14 |
| Ctg654_AF__Bc0067G15r | 17 | seryl-trna synthetase | chr14 |
| Ctg668_AF__Bc0060A24r | 17 | PREDICTED: hypothetical protein [Vitis vinifera] | chr14 |
| Ctg697_AF__Bb0026F15f | 17 | auxin efflux carrier component | chr14 |
| Ctg710_AF__Bb0017J01r | 17 | protein | chr14 |
| Ctg710_AF__Bc0032H13f | 17 | contains weak to the sapb protein (tr:e236624) | chr14 |
| Ctg764_AF__Bb0022B12f | 17 | glycogen synthase | chr14 |
| Ctg770_AF__Bc0068O21f | 17 | aldo keto | chr14 |
| Ctg809_AF__Bb0071L13f | 17 | protein | chr14 |
| Ctg920_AF__Bb0022I15f | 17 | acetolactate synthase | chr14 |
| Ctg934_AF__Bc0053L10f | 17 | pyruvate dehydrogenase kinase | chr14 |
| Ctg94_AF__Bc0001I21f | 17 | 40s ribosomal protein expressed | chr14 |
| Ctg964_AF__Bb0006E14f | 17 | homo copine i (gb | chr14 |
| Ctg994_AF__Bb0022A08r | 17 | n -dimethylguanosine trna | chr14 |
| Ctg1739_AF__Bc0075J15f | 10 | protein | chr14_random |
| Ctg270_AF__Bc0057N12r | 3 | senescence-associated family protein | chr14_random |
| Ctg303_AF__Bb0011E18r | 7 | protein | chr14_random |
| Ctg374_AF__Bc0062L01f | 4 | pyridoxal kinase-like protein | chr14_random |
| Ctg952_AF__Bb0012G09r | 17 | retrotransposon gag protein | chr14_random |
| Ctg1043_AF__Bb0054B13r | 11 | aminoimidazolecarboximide ribonucleotide transformylase inosine monophosphate cyclohydrolase | chr15 |
| Ctg127_AF__Bb0026B13f | 51 | phosphoribosyl pyrophosphate synthase | chr15 |
| Ctg217_AF__Bc0029O18f | 5 | transcriptional expressed | chr15 |
| Ctg225_AF__Bc0031L12r | 2 | protein | chr15 |
| Ctg2621_AF__Bb0004L19f | 109 | trna 2 | chr15 |
| Ctg2821_AF__Bc0039P15f | 32 | chalcone synthase family protein | chr15 |
| Ctg333_AF__Bc0070L10f | 3 | hypoersensitive response protein | chr15 |
| Ctg3557_AF__Bc0037L18r | 65 | ku p80 dna | chr15 |
| Ctg3758_AF__Bc0044K03r | 15 | chromatin remodeling complex subunit | chr15 |
| Ctg3852_AF__Bb0032O07r | 32 | transketolase-like protein | chr15 |
| Ctg4594_AF__Bb0042J02f | 2 | gag-pol identical | chr15 |
| Ctg641_AF__Bb0027G16f | 17 | nucleotide sugar epimerase | chr15 |
| Ctg917_AF__Bc0001B16f | 17 | integral membrane single c2 domain protein | chr15 |
| Ctg1227_AF__Bc0041G21f | 27 | protein | chr15_random |
| Ctg142_AF__Bb0053M12r | 5 | pentatricopeptide repeat-containing | chr15_random |
| Ctg1451_AF__Bb0020I02f | 8 | retrotransposon ty1-copia subclass | chr15_random |
| Ctg1476_AF__Bc0035J10r | 17 | translation initiation factor eif-2b epsilon | chr15_random |
| Ctg1711_AF__Bc0027F19r | 7 | hevamine-a | chr15_random |
| Ctg470_AF__Bc0027J15r | 2 | pre-mrna-splicing factor cwc-22 | chr15_random |
| Ctg506_AF__Bc0071M17r | 17 | beclin 1 protein | chr15_random |
| Ctg596_AF__Bb0034M20f | 17 | mitochondrial carrier protein cgi-69 | chr15_random |
| Ctg64_AF__Bb0001A19f | 17 | set domain protein | chr15_random |
| Ctg666_AF__Bb0006P02r | 17 | eukaryotic translation initiation factor 3 | chr15_random |
| Ctg1031_AF__Bb0054M10r | 14 | vacuolar atp synthase subunit | chr16 |
| Ctg1089_AF__Bc0016O09f | 10 | oligopeptide transporter opt family | chr16 |
| Ctg1110_AF__Bc0044L20f | 20 | alats (alanyl-trna synthetase) atp binding alanine-trna ligase forming aminoacyl-trna and related compounds nucleic acid binding nucleotide binding | chr16 |
| Ctg1162_AF__Bb0039N08f | 19 | gag-pol polyprotein | chr16 |
| Ctg1263_AF__Bc0056G24r | 22 | protein | chr16 |
| Ctg1303_AF__Bc0006D21f | 30 | alpha beta fold family protein | chr16 |
| Ctg142_AF__Bb0052D10f | 5 | nadh-dependent glutamate synthase | chr16 |
| Ctg187_AF__Bb0057M07f | 3 | pentatricopeptide repeat-containing | chr16 |
| Ctg26_AF__Bb0065O18f | 2 | ac055769_6copia-type 28768-32772 | chr16 |
| Ctg3802_AF__Bb0062J16f | 2 | nadh-dependent glutamate synthase | chr16 |
| Ctg460_AF__Bb0048I19f | 2 | protein | chr16 |
| Ctg53_AF__Bc0063K16r | 17 | protein | chr16 |
| Ctg599_AF__Bc0069E08r | 17 | wd40-repeat protein | chr16 |
| Ctg683_AF__Bc0063M06r | 17 | cytochrome p450 | chr16 |
| Ctg1291_AF__Bb0015P13r | 15 | PREDICTED: hypothetical protein [Vitis vinifera] | chr16_random |
| Ctg1313_AF__Bc0060P16r | 14 | protein | chr16_random |
| Ctg225_AF__Bb0030K13f | 2 | ---NA--- | chr16_random |
| Ctg225_AF__Bc0031L12f | 2 | ---NA--- | chr16_random |
| Ctg3596_AF__Bc0071D17r | 2 | gtp-binding protein | chr16_random |
| Ctg3786_AF__Bb0048N06r | 4 | protein | chr16_random |
| Ctg4332_AF__Bb0048K07f | 33 | protein | chr16_random |
| Ctg541_AF__Bc0072E02f | 17 | copia-like polyprotein | chr16_random |
| Ctg543_AF__Bc0030F22r | 17 | enolase | chr16_random |
| Ctg543_AF__Bc0030M19f | 17 | protein | chr16_random |
| Ctg605_AF__Bc0026I19f | 17 | apocytochrome b | chr16_random |
| Ctg1002_AF__Bb0073L20r | 14 | phosphatidyl serine synthase family protein | chr17 |
| Ctg1053_AF__Bb0070B11r | 52 | probable 26s proteasome regulatory subunit | chr17 |
| Ctg1064_AF__Bb0007E03f | 20 | phosphoglycerate bisphosphoglycerate mutase family protein | chr17 |
| Ctg1075_AF__Bc0050K14r | 32 | afc1 (arabidopsis fus3-complementing gene 1) atp binding kinase protein kinase protein serine threonine kinase protein tyrosine kinase | chr17 |
| Ctg1304_AF__Bc0013N11f | 15 | replication factor c dna polymerase iii gamma-tau | chr17 |
| Ctg1356_AF__Bc0064J03f | 26 | protein | chr17 |
| Ctg1459_AF__Bc0036G05r | 14 | cytochrome p450 | chr17 |
| Ctg1914_AF__Bc0038G23r | 6 | beta chain | chr17 |
| Ctg1925_AF__Bb0042J10r | 7 | replication factor a | chr17 |
| Ctg2041_AF__Bb0013G08r | 5 | ---NA--- | chr17 |
| Ctg2343_AF__Bc0010M09r | 6 | protein | chr17 |
| Ctg2619_AF__Bc0052E06r | 9 | protein | chr17 |
| Ctg2654_AF__Bb0054N14r | 12 | protein kinase | chr17 |
| Ctg30_AF__Bc0004A07f | 7 | protein expressed | chr17 |
| Ctg3021_AF__Bb0035K23r | 3 | protein | chr17 |
| Ctg313_AF__Bc0037H17r | 2 | protein | chr17 |
| Ctg365_AF__Bc0069B02f | 2 | transitional endoplasmic reticulum | chr17 |
| Ctg385_AF__Bc0020C18r | 6 | comm domain-containing | chr17 |
| Ctg3924_AF__Bc0011P04f | 27 | brain protein 44-like protein | chr17 |
| Ctg4017_AF__Bb0007L13r | 5 | pentatricopeptide repeat-containing | chr17 |
| Ctg4065_AF__Bc0040A16f | 27 | at5g03860 | chr17 |
| Ctg4208_AF__Bb0069I17f | 50 | light-harvesting complex i protein lhca5 | chr17 |
| Ctg445_AF__Bb0011L13r | 4 | at1g18090 t10f20_6 | chr17 |
| Ctg486_AF__Bc0052L18r | 17 | retrotransposon unclassified | chr17 |
| Ctg54_AF__Bc0064E10r | 17 | peroxidase 12 | chr17 |
| Ctg567_AF__Bc0035F13r | 17 | protein | chr17 |
| Ctg59_AF__Bc0055L07f | 17 | polyprotein 1 | chr17 |
| Ctg646_AF__Bc0065M02f | 17 | ---NA--- | chr17 |
| Ctg668_AF__Bb0051H05r | 17 | ---NA--- | chr17 |
| Ctg69_AF__Bb0027D13r | 17 | heat shock | chr17 |
| Ctg802_AF__Bb0023J15r | 17 | ---NA--- | chr17 |
| Ctg93_AF__Bc0018K01r | 17 | adenosylhomocysteinase s-adenosyl-l-homocysteine hydrolase | chr17 |
| Ctg931_AF__Bb0005I08r | 17 | histone acetyltransferase | chr17 |
| Ctg977_AF__Bb0065E15r | 17 | ---NA--- | chr17 |
| Ctg1065_AF__Bc0025P09r | 9 | major facilitator superfamily protein | chr17_random |
| Ctg1563_AF__Bc0046H10f | 13 | major facilitator superfamily protein | chr17_random |
| Ctg1658_AF__Bb0043A15f | 6 | hypothetical protein [Vitis vinifera] | chr17_random |
| Ctg546_AF__Bc0001H12f | 17 | major facilitator superfamily protein | chr17_random |
| Ctg546_AF__Bc0057F17f | 17 | major facilitator superfamily protein | chr17_random |
| Ctg643_AF__Bc0017A16r | 17 | predicted protein [Populus trichocarpa] | chr17_random |
| Ctg1059_AF__Bb0013P04r | 28 | protein | chr18 |
| Ctg1149_AF__Bc0039P05f | 14 | protein kinase-like protein | chr18 |
| Ctg1185_AF__Bc0029L24r | 9 | dna double-strand break repair rad50 | chr18 |
| Ctg12_AF__Bc0063H10f | 34 | protein | chr18 |
| Ctg12_AF__Bc0070B11f | 34 | receptor serine-threonine protein | chr18 |
| Ctg1220_AF__Bc0058L03f | 9 | protein | chr18 |
| Ctg132_AF__Bc0063O11r | 5 | copia-type polyprotein | chr18 |
| Ctg133_AF__Bc0058L16f | 4 | protein | chr18 |
| Ctg1408_AF__Bc0022B23r | 16 | ---NA--- | chr18 |
| Ctg1528_AF__Bc0034L09f | 10 | retrotransposon ty3-gypsy subclass | chr18 |
| Ctg1561_AF__Bc0039J19r | 8 | serine carboxypeptidase | chr18 |
| Ctg1566_AF__Bc0009A04r | 5 | ap2 erf domain-containing transcription factor | chr18 |
| Ctg1596_AF__Bb0017L21r | 37 | kinesin-like protein | chr18 |
| Ctg1693_AF__Bb0019D03r | 7 | atp binding | chr18 |
| Ctg1733_AF__Bb0006J20r | 7 | gag-protease-integrase-rt-r polyprotein | chr18 |
| Ctg1736_AF__Bb0010G04f | 9 | cop8 (constitutive photomorphogenic) homolog | chr18 |
| Ctg181_AF__Bc0046E09r | 5 | mynd finger family expressed | chr18 |
| Ctg193_AF__Bb0035G12r | 6 | protein | chr18 |
| Ctg193_AF__Bb0047F06f | 6 | replication factor c 36kda subunit | chr18 |
| Ctg193_AF__Bc0002N18r | 6 | ---NA--- | chr18 |
| Ctg197_AF__Bc0020J01f | 7 | antioxidant oxidoreductase | chr18 |
| Ctg2110_AF__Bb0047P15r | 9 | inositol polyphosphate 5-phosphatase | chr18 |
| Ctg2110_AF__Bc0055B10f | 9 | inositol polyphosphate 5-phosphatase | chr18 |
| Ctg215_AF__Bb0029A19f | 15 | cryptochrome 1 | chr18 |
| Ctg233_AF__Bb0025J07f | 2 | dna binding | chr18 |
| Ctg233_AF__Bc0024A14f | 2 | aberrant pollen transmission 1 | chr18 |
| Ctg242_AF__Bb0043K20r | 3 | atp binding | chr18 |
| Ctg245_AF__Bb0075B09f | 3 | gag-pol polyprotein | chr18 |
| Ctg249_AF__Bb0033P11r | 5 | protein | chr18 |
| Ctg2518_AF__Bc0074F10r | 7 | wd and tetratricopeptide repeat | chr18 |
| Ctg255_AF__Bb0012C04f | 2 | splicing endonuclease positive effector | chr18 |
| Ctg255_AF__Bb0023M01f | 2 | sulphate transporter | chr18 |
| Ctg2558_AF__Bb0031F02f | 7 | ---NA--- | chr18 |
| Ctg2597_AF__Bb0045H16f | 4 | ---NA--- | chr18 |
| Ctg2821_AF__Bc0039P15r | 32 | protein | chr18 |
| Ctg306_AF__Bb0069H06r | 2 | protein | chr18 |
| Ctg3490_AF__Bc0058K10r | 42 | iaa-amino acid hydrolase ilr1 | chr18 |
| Ctg367_AF__Bb0022D01f | 2 | af369930_2pol polyprotein | chr18 |
| Ctg3766_AF__Bb0011B05f | 36 | family with sequence similarity member a1 | chr18 |
| Ctg3782_AF__Bb0015K10r | 26 | protein | chr18 |
| Ctg3817_AF__Bb0061K17r | 65 | protein | chr18 |
| Ctg382_AF__Bb0048N17r | 34 | endo- -beta-glucanase | chr18 |
| Ctg4264_AF__Bc0052O11r | 6 | polynucleotidyl ribonuclease h fold | chr18 |
| Ctg431_AF__Bb0007B23r | 4 | kinesin-like protein | chr18 |
| Ctg4332_AF__Bc0027H10f | 33 | vacuolar protein sorting-associated protein | chr18 |
| Ctg4409_AF__Bb0063M17r | 43 | protein | chr18 |
| Ctg460_AF__Bc0029O07r | 2 | protein | chr18 |
| Ctg464_AF__Bb0050G24r | 20 | chlorophyll a b-binding protein | chr18 |
| Ctg468_AF__Bc0042B01f | 2 | hypothetical protein OsJ_08481 [Oryza sativa Japonica Group] | chr18 |
| Ctg471_AF__Bb0034O05f | 42 | predicted protein [Populus trichocarpa] | chr18 |
| Ctg520_AF__Bc0052O15f | 17 | mediator of rna polymerase ii transcription subunit 18 | chr18 |
| Ctg524_AF__Bc0017I23f | 17 | cytochrome p450 like_tbp | chr18 |
| Ctg533_AF__Bb0050E20f | 17 | protein | chr18 |
| Ctg580_AF__Bc0011G12f | 17 | chromatin binding | chr18 |
| Ctg586_AF__Bc0060D22r | 17 | ptac6 (plastid transcriptionally active6) | chr18 |
| Ctg624_AF__Bc0064B07r | 17 | ---NA--- | chr18 |
| Ctg678_AF__Bc0003I19r | 17 | protein kinase | chr18 |
| Ctg681_AF__Bb0071H13r | 17 | alcohol dehydrogenase | chr18 |
| Ctg683_AF__Bb0046D17r | 17 | serine threonine protein kinase pbs1 | chr18 |
| Ctg708_AF__Bc0031G20f | 17 | emb1967 (embryo defective 1967) | chr18 |
| Ctg733_AF__Bc0006N15f | 17 | protein | chr18 |
| Ctg756_AF__Bb0067H02r | 17 | o- c transferase | chr18 |
| Ctg771_AF__Bc0074B01f | 17 | rna polymerase ii largest subunit | chr18 |
| Ctg814_AF__Bb0064P13f | 17 | protein | chr18 |
| Ctg82_AF__Bb0063F13r | 17 | squalene monooxygenase | chr18 |
| Ctg827_AF__Bc0031G23r | 17 | receptor protein kinase clavata1 | chr18 |
| Ctg885_AF__Bc0035K14f | 17 | inositol-tetrakisphosphate 1- | chr18 |
| Ctg967_AF__Bc0022D13r | 17 | acetyl- carboxylase | chr18 |
| Ctg1745_AF__Bb0034B06r | 21 | at1g43690 f2j6_4 | chr18_random |
| Ctg1745_AF__Bc0048J24r | 21 | protein | chr18_random |
| Ctg1914_AF__Bc0073I11f | 6 | nucleolar autoantigen-like protein | chr18_random |
| Ctg277_AF__Bb0065F18r | 3 | fructose- -bisphosphatase | chr18_random |
| Ctg306_AF__Bb0050P16f | 2 | axi 1 protein from nicotiana tabacum | chr18_random |
| Ctg3549_AF__Bb0042H04r | 12 | protein | chr18_random |
| Ctg3583_AF__Bc0048N14f | 44 | cellulose synthase catalytic subunit | chr18_random |
| Ctg3698_AF__Bc0040E13f | 21 | non-imprinted in prader-willi angelman syndrome region | chr18_random |
| Ctg3766_AF__Bb0039K14r | 36 | histidinol-phosphate aminotransferase | chr18_random |
| Ctg3766_AF__Bb0051K15f | 36 | calcium-dependent protein kinase | chr18_random |
| Ctg493_AF__Bb0052P15r | 17 | gtk16 protein | chr18_random |
| Ctg1143_AF__Bb0017L20f | 17 | protein | chr19 |
| Ctg129_AF__Bc0009C05f | 39 | protein | chr19 |
| Ctg1337_AF__Bc0027D05f | 7 | glucose-6-phosphate translocator | chr19 |
| Ctg1353_AF__Bb0048M06f | 8 | predicted protein [Populus trichocarpa] | chr19 |
| Ctg1386_AF__Bc0001H02f | 20 | fimbrin 1 | chr19 |
| Ctg1452_AF__Bc0016C08f | 14 | cytochrome | chr19 |
| Ctg1460_AF__Bc0063N17f | 13 | ---NA--- | chr19 |
| Ctg1533_AF__Bb0076N19f | 8 | ---NA--- | chr19 |
| Ctg159_AF__Bc0047M07f | 5 | unnamed protein product [Vitis vinifera] | chr19 |
| Ctg1706_AF__Bb0030I03f | 11 | 4-methyl-5(b-hydroxyethyl)-thiazol monophosphate biosynthesis enzyme | chr19 |
| Ctg1707_AF__Bc0002J20r | 7 | multidrug resistance protein | chr19 |
| Ctg1739_AF__Bc0048L01r | 10 | protein | chr19 |
| Ctg2005_AF__Bc0003E22f | 6 | protein | chr19 |
| Ctg2005_AF__Bc0003E22r | 6 | protein | chr19 |
| Ctg21_AF__Bc0060P18f | 41 | choline transporter-related | chr19 |
| Ctg239_AF__Bc0025M15r | 3 | protein | chr19 |
| Ctg2458_AF__Bb0062I11f | 5 | ubc13a ubc35 (ubiquitin-conjugating enzyme35) protein binding ubiquitin-protein ligase isoform 2 | chr19 |
| Ctg317_AF__Bb0010C05r | 4 | atp binding | chr19 |
| Ctg33_AF__Bb0051O05f | 22 | ---NA--- | chr19 |
| Ctg341_AF__Bc0045K04f | 2 | cytochrome p450 | chr19 |
| Ctg3675_AF__Bc0039M21f | 31 | gtp-binding protein | chr19 |
| Ctg3744_AF__Bb0039I06f | 48 | nucleic acid binding protein | chr19 |
| Ctg3744_AF__Bc0018C07r | 48 | proteasome activator subunit 4-like | chr19 |
| Ctg394_AF__Bb0009I12r | 24 | beta-amylase | chr19 |
| Ctg400_AF__Bc0043P01f | 10 | ubiquitin | chr19 |
| Ctg4227_AF__Bc0008K14f | 16 | cytochrome | chr19 |
| Ctg472_AF__Bc0056N16r | 10 | wd-40 repeat family protein | chr19 |
| Ctg486_AF__Bb0077A12f | 17 | protein | chr19 |
| Ctg555_AF__Bb0042F04f | 17 | protein binding | chr19 |
| Ctg557_AF__Bc0047K21f | 17 | mitochondrial translational initiation | chr19 |
| Ctg56_AF__Bc0072A24f | 17 | protein | chr19 |
| Ctg581_AF__Bb0041G06r | 17 | protein | chr19 |
| Ctg701_AF__Bc0018L16r | 17 | protein | chr19 |
| Ctg717_AF__Bb0040L13r | 17 | peptide-n4-(n-acetyl-beta-glucosaminyl)asparagine amidase | chr19 |
| Ctg738_AF__Bb0050M14f | 17 | protein | chr19 |
| Ctg738_AF__Bc0031I09r | 17 | ---NA--- | chr19 |
| Ctg74_AF__Bc0025M10r | 17 | flowering locus d | chr19 |
| Ctg769_AF__Bb0016K14f | 17 | cdp-diacylglycerol synthetase | chr19 |
| Ctg776_AF__Bb0034K01r | 17 | vacuolar sorting protein | chr19 |
| Ctg778_AF__Bb0060E22r | 17 | solanesyl diphosphate synthase | chr19 |
| Ctg800_AF__Bb0069H14r | 17 | multidrug resistance protein abc transporter family | chr19 |
| Ctg915_AF__Bb0020O22f | 17 | protein | chr19 |
| Ctg931_AF__Bb0008D03r | 17 | ---NA--- | chr19 |
| Ctg3731_AF__Bb0068J12f | 3 | retrotransposon ty3-gypsy subclass | chr19_random |
| Ctg4333_AF__Bb0063K12r | 14 | transferring glycosyl | chr19_random |
